# Effectiveness of medication review: a systematic review and meta-analysis of randomized controlled trials

**DOI:** 10.1186/s12875-016-0577-x

**Published:** 2017-01-17

**Authors:** Victor Johan Bernard Huiskes, David Marinus Burger, Cornelia Helena Maria van den Ende, Bartholomeus Johannes Fredericus van den Bemt

**Affiliations:** 1 Department of Pharmacy, Sint Maartenskliniek, Hengstdal 3, 6574 NA Ubbergen, The Netherlands; 2 Department of Pharmacy, Radboud University Medical Center, Geert Grooteplein-Zuid 10, 6525 GA Nijmegen, The Netherlands; 3 Department of Rheumatology, Sint Maartenskliniek, Hengstdal 3, 6574NA Ubbergen, The Netherlands; 4 Department of Clinical Pharmacy and Toxicology, Maastricht University Medical Center +, Maastricht, Peter Debyelaan 15, 6229 HX Maastricht, The Netherlands

**Keywords:** Medication review, Drug utilization review[Mesh], Pharmaceutical services[Mesh], Review[Publication Type], Meta-analysis[Publication Type], Clinical outcomes, Quality of life, Drug-related outcomes, Economical outcomes

## Abstract

**Background:**

Medication review is often recommended to optimize medication use. In clinical practice it is mostly operationalized as an intervention without co-interventions during a short term intervention period. However, most systematic reviews also included co-interventions and prolonged medication optimization interventions. Furthermore, most systematic reviews focused on specific patient groups (e.g. polypharmacy, elderly, hospitalized) and/or on specific outcome measures (e.g. hospital admissions and mortality). Therefore, the objective of this study is to assess the effectiveness of medication review as an isolated short-term intervention, irrespective of the patient population and the outcome measures used.

**Methods:**

A literature search was performed in MEDLINE, EMBASE and Web of Science from their inception through September 2015. Randomized controlled trials (RCTs) with medication review as isolated short term intervention (<3 months) were included. There were no restrictions with regard to patient characteristics and outcome measures. One reviewer extracted and a second checked data. The risk of bias of studies was evaluated independently by two reviewers. A best evidence synthesis was conducted for every outcome measure used in more than one trial. In case of binary variables a meta-analysis was performed in addition to the best evidence synthesis, to quantify the effect.

**Results:**

Thirty-one RCTs were included in this systematic review (55% low risk of bias). A best evidence synthesis was conducted for 22 outcome measures. No effect of medication review was found on clinical outcomes (mortality, hospital admissions/healthcare use, the number of patients falling, physical and cognitive functioning), except a decrease in the number of falls per patient. However, in a sensitivity analysis using a more stringent threshold for risk of bias, the conclusion for the effect on the number of falls changed to inconclusive. Furthermore no effect was found on quality of life and evidence was inconclusive about the effect on economical outcome measures. However, an effect was found on most drug-related problems: medication review resulted in a decrease in the number of drug-related problems, more changes in medication, more drugs with dosage decrease and a greater decrease or smaller increase of the number of drugs.

**Conclusions:**

An isolated medication review during a short term intervention period has an effect on most drug-related outcomes, minimal effect on clinical outcomes and no effect on quality of life. No conclusion can be drawn about the effect on economical outcome measures. Therefore, it should be considered to stop performing cross-sectional medication reviews as standard care.

**Electronic supplementary material:**

The online version of this article (doi:10.1186/s12875-016-0577-x) contains supplementary material, which is available to authorized users.

## Background

In order to reduce the number of preventable adverse drug events and hospital admissions, medication review is often recommended, incorporated in several guidelines and also frequently reimbursed by health care insurers in various countries [1–10]. Medication review is defined by the Pharmaceutical Care Network Europe (PCNE) as “a structured evaluation of a patient‘s medicines with the aim of optimising medicines use and improving health outcomes. This entails detecting drug related problems and recommending interventions” [11]. In clinical practice, for each individual patient, medication review is mostly operationalized as an isolated intervention during a short term intervention period [5, 6, 9, 12, 13].

Several systematic reviews and meta-analyses already examined the effectiveness of medication review and these did not unequivocally prove the effectiveness of medication review [14–23]. However, these systematic reviews did not only include trials assessing the effect of medication review in terms of how it is mostly operationalized in practice: an isolated cross-sectional assessment of total medication use during a short term intervention period less than 3 months. Most trials in the systematic reviews assessed the effect of medication review as part of multi-faceted pharmaceutical care interventions, consisting of for instance transitional care, adherence counseling and education of patients and healthcare professionals, besides medication review. Such interventions also often last longer than 3 months. Furthermore, most systematic reviews focus on specific patient groups (e.g. polypharmacy, elderly, hospitalized) and/or on specific outcome measures (e.g. hospital admissions and mortality). As a result, more insight is necessary in the effectiveness of medication review as an isolated short-term intervention on clinical outcomes, quality of life, drug-related and economical outcomes.

Therefore, this systematic review aims to summarize the evidence of medication reviews as performed in clinical practice, irrespective of patient characteristics, setting and outcome measures.

## Methods

This systematic review, assessing the effectiveness of medication review, irrespective of the outcome measures used, follows the PRISMA-guidelines [24, 25].

### Data sources and searches

A literature search was performed in MEDLINE, EMBASE and Web of Science from their inception through September 2015. For the development of the search strategy and the full electronic search, see Additional file 1.

### Study selection

The inclusion criteria were operationalized based on the PICO model. No restrictions were set concerning the P (patients) and O (outcome measures): interventions could be conducted in any setting and there were no restrictions with regard to patient characteristics and outcome measures. The I (intervention) had to be medication review, which was defined as follows: a structured cross-sectional assessment of a patient’s total medication use leading to recommendations that had to be discussed with the patient and/or clinician within 3 months, in order to improve safety, efficacy or cost-effectiveness. Medication review had to be the single intervention; co-interventions with potential impact on the outcome measures (e.g. discharge counseling, transitional care, non-pharmacological interventions) were not allowed. The C (comparison) was defined as usual care. In addition to PICO the following study selection criteria were formulated: trials had to be randomized controlled trials (RCTs) and only full-length articles were considered for inclusion in this review. Two reviewers independently selected titles/abstracts and the corresponding full text articles to be included in this systematic review. Discrepancies in judgment were discussed in order to reach consensus (VH-BvdB) about final inclusion.

### Data extraction and risk of bias assessment

Relevant data on study characteristics and outcomes were extracted by one reviewer (VH) and checked by a second reviewer (NW). *P*-values ≤0.05 were considered as statistically significant.

Two reviewers independently assessed the risk of bias of the studies eligible for inclusion by using the checklist with criteria for risk of bias from the Cochrane Back Review Group [26, 27]. To determine whether a study had a low risk of bias (LRB) or a high risk of bias (HRB), a consensus (VH-BvdB) based scorings method was developed based on the risk of bias assessment.

The twelve Cochrane criteria [26, 27] were designated essential (4) or non-essential (8) in relation to research on medication review by a consensus discussion (VH-BvdB). Essential criteria were: was the method of randomization adequate?; Was the drop-out rate described and acceptable?; Were the groups similar at baseline regarding the most important prognostic indicators?; Were co-interventions avoided or similar?. To be considered a study with a low risk of bias, all the essential Cochrane criteria had to be scored positive, whereas a total of at least 6 of the 12 criteria (50%) had to be scored positive. A cutoff of 50% was chosen, as it is not feasible for medication review trials to score positive on certain criteria, like: “was the patient blinded to the intervention”; “was the care provider blinded to the intervention”; “was the outcome assessor blinded to the intervention”. Discrepancies in judgment were discussed in order to reach consensus (VH-BvdB) about the designation of low or high risk of bias for each criterion for each study. If for a specific study an “unclear risk of bias” was scored for the same criterion by both reviewers, the criterion was designated “high risk of bias. The inter-rater agreement of the assessment of risk of bias was assessed by calculating the Cohen’s kappa.

A sensitivity analysis was performed regarding a more stringent cut-off point for risk of bias. The actually used cut-off point for risk of bias was compared with a threshold of ≥8 (2/3 of the attainable 12) of the criteria to be scored positive for a study to be considered a study with a low risk of bias.

### Data synthesis and analysis

An adapted version from previously published best evidence syntheses [28, 29] was conducted for every outcome measure used in more than one trial, combining a) the percentage of intervention patients included in studies showing effect on the outcome measure and b) the risk of bias of the set of trials using the outcome measure.

**Fig. 1:**
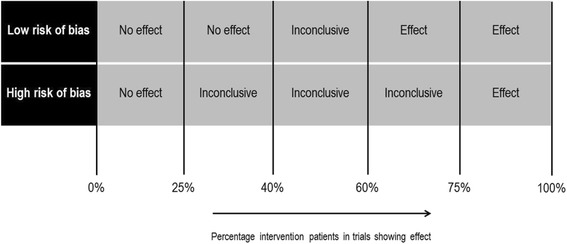
Schematic representation of the best evidence synthesis. Schematic representation of the best evidence synthesis, combining **a**) the percentage of intervention patients included in studies showing effect on the outcome measure and **b**) the risk of bias of the set of trials using the outcome measure. For details: see Additional file 4

The following methodology was used for this purpose:

1. First, for each outcome measure, the percentage of intervention patients included in studies showing effect on the outcome measure was calculated
2. The risk of bias of a set of studies per outcome measure was subsequently determined as follows: if 50% or more of the intervention patients included in trials using the outcome measure had a low risk of bias, the set of studies was designated overall low risk of bias
3. Finally, both the percentage of intervention patients included in studies showing effect on the outcome measure and the risk of bias score for the set of trials per outcome measure were combined to conclude whether medication review has effect on the outcome measure by using the method depicted in Fig. 1.

In case of binary variables a meta-analysis was performed in addition to the best evidence synthesis, to quantify the effect. In these meta-analyses, effect sizes of binary variables were pooled using their weighted average for the treatment effect (using a random-effect meta-analysis method). Forest plots were created with STATA version 13.1 to summarize the risk ratio (RR) and the 95% confidence interval (CI). Heterogeneity across studies was assessed using I^2^ statistics (studies with an I^2^ > 50% were considered heterogeneous). Outcome measures reported in only one trial were reported descriptively.

A sensitivity analysis was performed with regard to the impact of large trials with a high risk of bias, on every individual outcome measure. In this sensitivity analysis, large trials with a high risk of bias, with a number of intervention patients greater than the median number of intervention patients per outcome measure, were excluded from the best evidence synthesis.

## Results

**Fig. 2:**
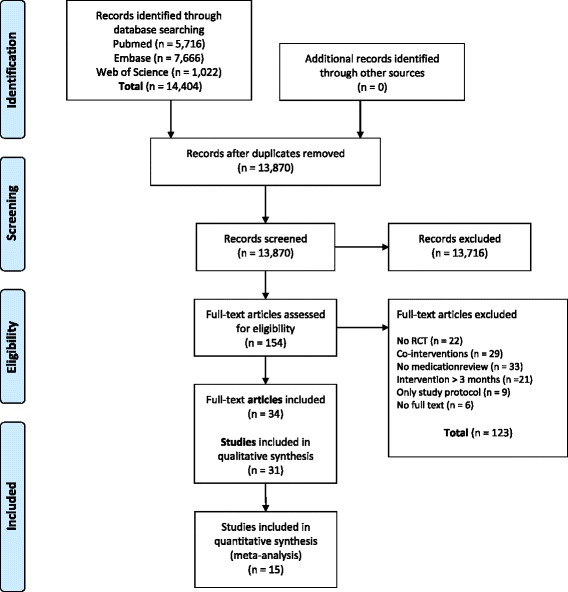
Flow diagram of the literature search and study selection process

The literature search provided a total of 13,870 potentially relevant publications which were screened for eligibility. After screening titles and abstracts, 154 articles were left for full text screening. After this screening, 31 RCTs met the inclusion criteria and were included in this systematic review. A flow diagram of the literature search is represented in Fig. 2.

An overview of the study characteristics of the included studies is depicted in Table 1. Most studies were conducted in primary care (52%), sample size ranged from 64 to 2014 patients, the observation period ranged from 0 to 12 months and 17 (58%) of the trials were conducted in Europe and 7 (23%) in the United States. Patients were involved in the medication reviews in 21 of the 31 studies, the mean age (as reported in 24/31 trials) and the number of drugs (as reported in 18/31 studies) of the intervention patients in each trial ranged from 51.4 to 87.0 years and from 4 to 14 drugs, respectively.

**Table 1:** Study characteristics of the included studies

Author (Year)	Risk of bias	Foll-ow up (mos.)	Country and setting	Mean age (IG), years	No. Pts.	Description intervention				Patient selection criteria for medication review		
						HCP involvement R: Medication review D: Decision about clinical relevancy	Patient Involve-ment	Nr assess-ments/nr patient contacts	Additional Education HCP	Age, years	Nr drugs	Other
Bond [38] (2007)	LRB	12	GB, general practices	Nr	2014	R: Pharmacist D: GP	No	1	Yes	< 65 ^a^	No	Specific conditions^a^
Briggs [73] (2015)	HRB	4	AU, tertiary referral hospital	82.0	2015	R: Hospital pharmacist D: GP	Yes	1	Nr	>70^a^	>5^a^	Living at home^a^
Britton [51] (1991)	HRB	3	US, general medicine clinic	Nr	760	R: Clinical pharmacist D: physician (assistant)	No	1	Yes	No	> 5	No
Burns [32]/Furniss [42] (2000)	HRB	4	GB, nursing homes	83.5	330	R: (study) pharmacist D: multidisciplinary team	No	2	Nr	No	No	Living in nursing home
Gallagher [49] (2011)	LRB	6	GB, tertiary medical centre	74.5	400	R: (research) physician, medical team D: physician	No	1	Nr	≥ 65^a^	No	Emergency admission^a^
Graffen [74] (2004)	HRB	6	AU, general practices	Nr	402	R: Pharmacist D: GP and patient	Yes	1	Nr	> 65^a^	≥ 5^a^	Living independently^a^; ≥ 1 of following^a^: use of predefined risk drugs; > 12 doses per day; > 6 diagnoses; BMI < 22
Heselmans [33] (2015)	HRB	0^b^	BE, general and specialized hospitals	66.6	600	R: Pharmacist D: Ward physician	No	1	Nr	>15^a^	No	ICU stay of at least three consecutive day^a^
Holland [34] (2005)/Pacini [53] (2007)	LRB	6	GB, emergency wards	85.4	855	R: (study) pharmacist D: pharmacist or GP	Yes	2	Yes	> 80^a^	≥ 2^a^	Discharged after emergency admission to own home or warden controlled accommodation^a^
Jameson [47] (1995)	HRB	6	US, family health center	Nr	64	R: Clinical pharmacist D: Physician and pharmacist	Yes	2	Nr	No	≥ 5 (see other)	≥ 2 of following risk factors: ≥ 5 drugs; ≥ 12 daily doses; ≥ 4 medication changes last 12 months.; >3 concurrent diseases; noncompliance; drugs requiring TDM
Jameson [50] (2001)	HRB	6	US, private physicians	51.4	340	R: Clinical pharmacist D: Physician and pharmacist	Yes	1	Nr	No	≥ 5	No
Krska [75] (2001)	HRB	3	GB, medical practices	74.8	381	R: Clinical pharmacist D: GP and pharmacist	Yes	1	Nr	≥ 65^a^	≥ 4^a^	≥ 2 chronic conditions^a^
Kwint [76] (2011)	LRB	6	NL, community pharmacies	78.7	118	R: 2 research pharmacists D: GP and community pharmacist	No	1	Nr	≥ 65^a^	≥ 5^a^	living at home^a^; at least one drug had to be dispensed via an automated system^a^
Lenaghan [77] (2007)	HRB	6	GB, general practices	84.5	136	R: study-pharmacist D: GP and study- pharmacist	Yes	2	Nr	> 80^a^	≥ 4^a^	living in own homes^a^; ≥ 1 of following criteria^a^: living alone; confused mental state, vision or hearing impairment; prescribed medicines associated with medication-related morbidity; prescribed >7 regular oral medicines
Lenander [35] (2014)	HRB	12	SE, primary care centre	79.0	209	R: Geriatrics pharmacist D: GP and patient	Yes	1	No	> 65^a^	≥ 5^a^	already scheduled for an appointment with a GP^a^
Lim [41] (2004)	LRB	2	SG, geriatric outpatient clinic	79.6	126	R: pharmacist (of a pharmacist consult clinic) D: primary physician	Yes	1	Nr	No	> 3 (see other)	≥ 1 of following criteria: TDM required; polypharmacy (>3 drugs or >9 doses per day); non-compliance; self-administered drugs that require psychomotor skill and co-ordination; nasogastric tube feeding; >1 doctor managing care; hospitalized within the last 6 months.
Lisby [36] (2010)	LRB	3	DK, acute ward	80.2	100	R: Clinical pharmacist and a clinical pharmacologist D: ward physicians	Yes	2	Nr	≥ 70^a^	≥ 1^a^	expected to be admitted for more than 24 h^a^
Lisby [30] (2015)	LRB	3	DK, regional hospital	80.4	108	R: Clinical pharmacist and a clinical pharmacologist D: Orthopedic ward physicians	Yes	2	Nr	> 65^a^	≥ 4^a^	nonelective admission at orthopedic ward^a^; expected in-hospital length of stay (LOS) of a minimum of 24 hours^a^
Mannheimer [78] (2006)	LRB	6	SE, clinical internal medicine	71.0	305	P: nurse and clinical pharmacologist D: physician in charge	Yes	1	Nr	No	≥ 2^a^	patients who had been in hospital for < 24 h on Tue. to Fri. or for < 60 h on Mon. before a nurse screened the computerized medical record^a^
Meredith [45] (2002)	LRB	1.5	US, home care	80.3	317	P: nurse and clinical pharmacist D: Physician	Yes	1	Yes	≥ 65^a^	No	had ≥ 1 of the four possible study medication problems^a^; projected duration of home health care of ≥4 wks^a^
Meyer [79] (1991)	HRB	12	US, VAMC	Nr	312	R: study-physician (Group III, intensive intervention) D: Physicians and nurse practitioners	No	1	Nr	No	≥ 10	being followed by providers at the medical center
Michalek [39] (2014)	LRB	0^b^	DE, tertiary medical center	84^c^	114	R: Physicians D: Physicians	No	1	Nr	> 70^a^	≥ 3^a^	admitted to the acute geriatric unit^a^, stable health condition defined as no need for intermediate or intensive care unit treatment^a^, had at least three diseases in need for drug treatment^a^.
Milos [80] (2013)	LRB	2	SE, primary health care centres	87.0	374	R: Clinical pharmacist D: Physician	No	1	Yes	≥ 75^a^	No	users of the multi-dose drug dispensing system; living in nursing homes or their own homes with municipally provided home care
Olsson [46] (2012)	HRB	12	SE, primary care	83.4	150	R: study-physician D: Family physician	Yes	1	Nr	≥ 75^a^	≥ 5^a^	living in ordinary homes^a^
Pit [44] (2007)	HRB	12	AU, general practice	Nr	849	R: Doctors D: Doctors	Yes	1	Yes	≥ 65^a^	No	living in the community^a^
Pope [43] (2011)	LRB	6	GB, community hospitals	83.3	225	R: multidisciplinary panel D: General practitioner	No	1	Nr	No	No	permanent patients on the continuing-care wards
Sellors [52] (2001)	LRB	6	CA, family physician practice	76.4	132	R: study-pharmacist D: family physician	Yes	1	Yes	≥ 65^a^	≥ 4^a^	No
Sellors [37] (2003)	LRB	5	CAN, family physician practices	74.0	889	R: Pharmacist D: Physician	Yes	1	Nr	≥ 65^a^	≥ 5^a^	had been seen by their physician within; the past 12 months^a^; no evidence of cognitive impairment; could understand English.
Williams [40] (2004)	HRB	1.5	US, general medicine clinic	73.5	140	R: Interdisciplinary team (consultant pharmacist, physician and nurse) D: Primary physician	Yes	1	Nr	≥ 65^a^	≥ 5^a^	≥ 2 of the medications were potentially problematic drugs for common geriatric problems^a^; cognitively intact ^a^
Zermansky [48] (2001/2002)	LRB	12	GB, general practices	74.0	1188	R: Study-clinical pharmacist D: Pharmacist or GP	Yes	1	Nr	≥ 65^a^	≥ 1^a^	No
Zermansky [81] (2006)	LRB	6	GB, care homes	85.3	661	R: Study-clinical pharmacist D: GP	Yes	1	Nr	≥ 65^a^	≥ 1^a^	No
Zillich [31] (2014)	LRB	2	US, home health care centers	73.0	895	R: Pharmacist D: Patient, pharmacist, physician	Yes	3–4	Nr	No	No	All new patients admitted into Medicare’s defined 60-day home health care episode were eligible. Medicare eligibility for home health benefits requires ordering services by a physician who reviews the need for a patient’s care and certifies that the patient is homebound

*mos.* months, *IG* intervention group, *Pts.* patients, *HCP* healthcare professional, *LRB* low risk of bias, *HRB* high risk of bias, ^a^ combination of inclusion criteria (= “and”), *Nr* not reported, ^b^outcome measures determined directly after discharge from ICU and/or discharge from hospital, *TDM* therapeutic drug monitoring, *hr* hours, ^c^median, *VAMC* Veterans Affairs Medical Center

Seventeen studies (55%) met the criteria for low risk of bias. The inter-rater agreement between the two assessors of risk of bias was 0.74 (Cohen’s Kappa). Most common reasons for designating studies high risk of bias were methodological shortcomings on “compliance”, “treatment allocation concealment”, “blindness of patient, care provider and outcome assessor”, “randomization”, “similarity of study groups at baseline” and “drop-out rate”.

### Clinical outcomes

**Fig. 3:**
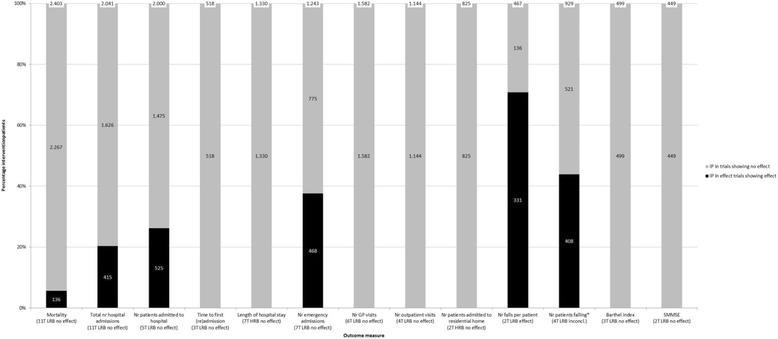
Effect of medication review on clinical outcome measures as assessed in more than 1 trial. The percentage of intervention patients is shown on the y-axis. The *black* part of the bar represents the percentage of intervention patients included in a trial with a positive effect on a specific outcome measure. The outcome measures, the number of trials using the specific outcome measure, the overall risk of bias of the set of evidence per outcome measure and the conclusion of the best evidence synthesis are shown on the x –axis. T = trials; LRB = low risk of bias; HRB = High risk of bias; inconcl. = inconclusive

As summarized in Fig. 3, no effect of medication review was found on clinical outcomes, except for a decrease in the number of falls.

#### Mortality

Eleven trials (overall low risk of bias, including 2403 intervention patients) assessed the effect of medication review on mortality (for details, see Additional file 2: Table S1). Data were pooled in a meta-analysis (Additional file 2: Figure S1) and with a RR of 0.94 (CI, 0.76–1.17) no effect of medication review on mortality was found. Moderate heterogeneity was found between the trials (I^2^ = 22.0%, *P* = 0.234).

#### Hospital admissions and healthcare use

Data of 11 trials (Additional file 2: Table S2), including 2041 intervention patients, showed evidence with a low risk of bias for no effect of medication review on the number of hospital admissions (including emergency admissions and visits). Meta-analysis of data from five trials with overall low risk of bias, including 2000 intervention patients, assessing the effect of medication review on the number of patients admitted to the hospital revealed no effect, with a RR of 0.94 (0.82, 1.08) and with moderate heterogeneity (I^2^ = 42.3%, *P* = 0.139) (Additional file 2: Figure S2 and Table S3). The same applies to the time to first (re)admission in three trials with low risk of bias, including 518 intervention patients, except for a subgroup with only emergency department visits or a low baseline risk for hospital admission (Additional file 2: Table S4) [30, 31]. In addition, no effect of medication review was found on the length of hospital stay in seven trials with overall high risk of bias, including 1330 intervention patients and the number of emergency admissions/visits in seven trials with overall low risk of bias, including 1243 intervention patients (Additional file 2: Tables S5 to S6). Furthermore, no effect of medication review was demonstrated on the number of General Practitioner (GP) visits in 6 trials with low risk of bias including 1582 intervention patients and on the number of outpatient visits in four trials with overall low risk of bias, including 1144 intervention patients (Additional file 2: Tables S7 to S8). The meta-analysis of data of 2 trials with overall high risk of bias, including 825 intervention patients, found no effect on the number of patients admitted to residential homes with a RR of 1.17 (0.79, 1.74), with limited heterogeneity (I^2^ = 0.0%, *p* = 0.997) (Additional file 2: Figure S3 and Table S9).

No best evidence synthesis could be conducted for a variety of other healthcare use related outcome measures used in only one trial. In six trials no effect was found on these outcome measures [30, 32–37], whereas in two trials an effect was found only in a subdomain of healthcare use related outcome measures or a subgroup of patients [32, 38] and in one trial a positive effect was found in favor of patients receiving usual care [30].

#### Falls

It was observed in two trials with overall low risk of bias, including 467 intervention patients, that medication review decreases the number of falls per patient (Additional file 2: Table S10). Data of four trials with overall low risk of bias, including 929 intervention patients, were pooled in a meta-analysis (Additional file 2: Figure S4). This meta-analysis suggested that medication review decreases the number of patients falling (RR 0.68 (0.52, 0.90); I^2^ = 41.0%, *p* = 0.166). However, the best evidence synthesis was inconclusive about the effect on the number of patients falling (Additional file 2: Table S11). Furthermore, a significant lower fall rate per 1000 patient days (only assessed by Michalek et al) due to medication review was found [39].

#### Health status, physical and cognitive outcome measures

Three trials with low risk of bias, including 499 intervention patients, showed no effect of medication review on physical functioning using the Barthel index (Additional file 2: Table S12). This was confirmed in one study, using three different outcome measures for physical functioning [40].

Medication review neither improved clinical status [41], health status [40] and patient’s perception of severity of illness [41]. In one study, however, a smaller decrease in self-rated health due to medication review was found [35].

Two trials, with overall low risk of bias, including 449 intervention patients, found no effect of medication review on cognitive functioning, using the Standard Mini Mental State Examination (Additional file 2: Table S13). Medication review also did not affect cognitive functioning, expressed with other outcome measures [40, 42, 43], except for the Chrichton-Royal Behaviour Rating Scale [42].

### Quality of life

**Fig. 4:**
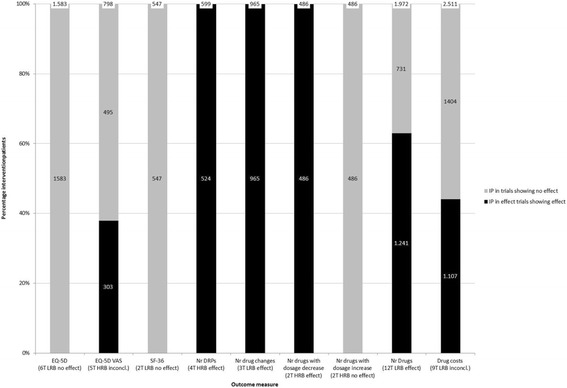
Effect of medication review on quality of life, drug-related outcome measures and economical outcome measures as assessed in more than one trial. The percentage of intervention patients is shown on the y-axis. The *black* part of the bar represents the percentage of intervention patients included in a trial with a positive effect on a specific outcome measure. The outcome measures, the number of trials using the specific outcome measure, the overall risk of bias of the set of evidence per outcome measure and the conclusion of the best evidence synthesis are shown on the x-axis. T = trials; LRB = low risk of bias; HRB = High risk of bias; inconcl. = inconclusive

The effect of medication review on quality of life is outlined in Fig. 4. There is evidence with overall low risk of bias that medication review has no effect on quality of life, as measured with the EQ-5D score (based on six trials, including 1583 intervention patients) or the SF-36 score (based on two trials, including 547 intervention patients), whereas evidence with overall high risk of bias was inconclusive about the effect of medication review on the EQ5D-VAS (used in five trials, including 798 intervention patients) (Additional file 2: Table S14). Pit et al also found no effect of medication review on quality of life measured with the SF-12 score [44].

### Drug-related outcome measures

The effect of medication review on drug-related outcome measures is represented in Fig. 4. An effect of medication review was found on most drug-related outcome measures (the number of drugs, the number of drug changes, the number of drug-related problems and the number of drugs with a dosage decrease), but not on the number of drugs with dosage increase.

#### Drug-related problems

In four trials with overall high risk of bias, including 599 intervention patients, medication review decreases the number of drug-related problems (Additional file 2: Table S15). The results of two trials assessing the effect of medication review on the number of patients with drug-related problems (with different pre-defined drug-related problems per trial) were conflicting [45, 46].

#### Number of drug changes and number of drugs with a dosage decrease or increase

Data of three trials with low risk of bias, including 965 intervention patients, showed an increase of the number of drug changes as a result of medication review (Additional file 2: Table S16). Two other trials with overall high risk of bias, including 486 intervention patients, found an increase of the number of drugs with a dosage decrease, whereas no difference was found with regard to the number of drugs with dosage increase (Additional file 2: Tables S17 to S18).

#### Number of drugs and doses

Twelve studies with overall low risk of bias, including 1972 intervention patients, found that medication review leads to a greater decrease or smaller increase of the number of drugs used (Additional file 2: Table S19). Sellors et al, however, found no difference in the absolute number of drugs used after 5 months due to medication review [37]. Furthermore, no effect of medication review was found on the number of individual doses per day [47] and the dosing frequency per day [48].

#### Other drug-related outcome measures

Various outcome measures, only used in one trial, but covering the same outcome domains, could not be incorporated in a best evidence synthesis. Two studies assessing the effect of medication review on adherence and knowledge found conflicting results [41, 47]. Results with regard to appropriate prescribing and medication use were also conflicting. In two trials, medication review did not improve a set of predefined indicators of prescription quality [44, 46], whereas other trials showed improvement of (part of) the indicators [38, 39, 49]. Trials reporting the effect of medication review on scores for appropriateness of prescribing and medication use also found conflicting results. Although medication review improved prescribing appropriateness as measured with the Medication Appropriateness Index (MAI) and the Assessment of Underutilization of Medication Index (AOU) [49], no effect was found on a composite score reflecting appropriate prescribing of benzodiazepines, NSAIDs and thiazide diuretics [44]. Finally, the effect of medication review on adverse effects was inconclusive, as one trial demonstrated that medication review decreases adverse effects [50] and a second trial did not show a significant effect [47].

### Economical outcomes

Figure 4 shows the effect of medication review on drug costs. Based on the data of nine trials with overall low risk of bias, including 2511 intervention patients, no conclusion could be drawn about the effect of medication review on drug costs (Additional file 2: Table S20). Trials using various other outcome measures for drug and supply costs did generally not observe effect of medication review on costs [37, 51, 52], except for one study demonstrating that medication review might decrease drug and supply costs due to discontinuation [51]. Inconclusive results were also observed with respect to total healthcare costs, as two studies found a positive effect of medication review on total healthcare costs [32, 53], one study found a temporary positive effect [38] and two studies did not find any effect [37, 43]. Besides this, Burns et al found no decrease or increase of costs related to non-drug GP visits, in patient days, outpatient visits, domiciliary visits and primary care visits due to medication review [32].

### Sensitivity analyses

The sensitivity analysis with a more stringent threshold for risk of bias (≥8; 2/3 of the attainable 12) yielded similar results except for the number of falls per patient, which changed from effective to inconclusive, see Additional file 3. Based on the sensitivity analysis excluding large trials with high risk of bias from the best evidence synthesis, twice the conclusion changed from effective to inconclusive (number of drug-related problems (DRPs) and number of drugs), twice from inconclusive to not effective (number of patients falling and drug costs), once from not effective to inconclusive (number of emergency admissions) and once from inconclusive to a decreased quality of life (EQ-5D VAS), see Additional file 4.

## Discussion

This is the first systematic review exploring the effect of medication review as an isolated intervention without co-interventions during a short term (≤3 months) intervention period (as advocated in most medication review guidelines [4–10] and operationalized in practice). Furthermore this systematic review provides an overview of all outcome measures and selection criteria without exclusion criteria based on patient characteristics. In this study, a beneficial effect of medication review was found on most drug-related outcome measures. However, minimal effect was observed on clinical outcomes, no effect was found on quality of life and evidence was inconclusive concerning the effect on economical outcome measures. Only seventeen trials (55%) were designated low risk of bias.

The findings of this systematic review are in line with the findings of other systematic reviews assessing the effect of medication review, although these systematic reviews used other inclusion criteria. Previously published systematic reviews often focused on specific patients (e.g. elderly or hospitalized patients etc.) and/or included trials with multifaceted interventions and/or limited the scope to specific outcome measures.

First of all, the lack of *effect of medication review on clinical outcomes* (e.g. mortality, number of hospital admissions) observed in this systematic review is in line with the findings of other systematic reviews [16–22], although Patterson found conflicting results concerning hospital admissions [14]. In other systematic reviews a positive effect of medication review on some clinical outcomes was suggested only when non RCTs [21], unpublished data [18], co-interventions [15, 18] and/or lengthier interventions (> 3 months) [21] were included. Secondly, no *effect of medication review on quality of life* was found by this systematic review, which is also confirmed by other systematic reviews [14, 16, 17, 21, 23]. Thirdly, the *effect of medication review on drug-related outcomes* (e.g. a decrease in the number of drug-related problems and the number of drugs) found in this systematic review was confirmed by other systematic reviews [17, 19], although Patterson found no consistent intervention effect on medication-related problems across studies [14]. In addition, in these systematic reviews an effect of medication review on some other drug-related outcome measures (e.g. adherence, adverse drug events, medication appropriateness) was reported [14–17, 19, 21, 23]. Finally, based on this systematic review, no conclusion could be drawn about the *effect of medication review on economical outcome measures*, including drug costs*.* These results were confirmed by the majority of other systematic reviews, since only one out of six other systematic reviews [23] reported effect of medication review on certain subdomains of economical outcome measures [15–17, 19, 21, 23].

Thus, when the effect of medication review is assessed in terms of how it is operationalized in practice (with medication review as isolated intervention) and even when this effect is assessed irrespective of the patient population and on all available outcome measures, the impact found on clinical outcomes and quality of life is minimal, the observed effect on drug-related outcomes is limited and the evidence about the effect on economical outcome measures is inconclusive. This requires further elaboration of the possible explanations of these findings. Several aspects seem to contribute to these findings, including the 1) *selection of patients*, the 2) *interventions* (how medication reviews are being operationalized in practice) and the 3) *outcome measures* and follow-up time used in trials assessing the effect of medication review. Besides these explanations it might also be the case that the hypothesis that medication review significantly improves clinical outcomes, economical outcomes and quality of life should be rejected.

A possible explanation for the lack of evidence about the effect of medication review is that the *1) selection of patients* does not fit the aim of the intervention. If the aim of medication review is, for example, decreasing mortality or preventing patients from being admitted to the hospital, one should select a population with high risk for any of these events. Inclusion criteria often mentioned in medication review trials are age 65-plus and a minimum number of drugs used. Although age and polypharmacy are predominantly positively associated with the risk of having drug-related problems [54–59], several other risk factors (e.g. co-morbidity, renal impairment, high risk medication) contributing to the occurrence of DRPs and/or hospital admissions are found in literature [54, 60–69]. This suggests that a more sensitive selection of patients for medication review in order to reduce the risk of hospital admission and or death may increase the chance of demonstrating an effect of medication review on these outcomes. Consequently, another aim of the intervention (e.g. increasing adherence) will require a different selection of patients (e.g. lack of therapeutic effect, adherence scores). A second explanation for the lack of evidence about the effect of medication review might be the heterogeneity of *2) the interventions*. No golden standard exists for how medication review should be operationalized in practice. Several implicit as well as explicit medication review methods are used [70]. Furthermore, different levels of medication review are applied in daily practice [10]. This limits the ability to compare the results of trials assessing the effect of medication review. In addition, the multidisciplinary character of medication reviews is possibly a complicating factor. Often problems are difficult to solve 1) as many care-practitioners are involved and 2) as it is not always clear which healthcare practitioner should be addressed and/or 3) as the responsible physician may not agree with implementation of a recommendation made by another healthcare practitioner. Once the aims of medication review are known, one or more consistent (international) definitions and accompanying operationalizations of medication review should be put into practice. Uniform medication reviews are easier to compare in systematic reviews, this will contribute to the ability to demonstrate effect of these interventions. Finally, the lack of evidence about the effect of medication review might be explained by *3) the outcome measures and follow-up time* used in trials assessing the effect of medication review. The outcome measures used in published RCTs examining the effect of medication review are often broad outcome measures, as for instance hospital admissions and all-cause mortality, which are affected by multiple (also not drug related) factors. Although in RCTs these outcome measures may be the ideal outcome measures, since these reflect the overall benefit/risk ratio of drug treatment, no effect of medication review on these outcome measures is found, possibly because the intervention medication review is not powerful enough to have impact on hospitalizations and mortality. Therefore (clinical) outcome measures should be chosen which fit 1) the aim of the medication review (improve safety and (cost-)effectiveness of a patient’s medication use) and 2) are more disease/medication specific (e.g. blood pressure, HbA1c) [12, 71]. However, these more disease/medication specific outcome measures should not only reflect the negative effects, but also the positive effects of drug treatment. Although it is often seen in medication review trials, only reporting drug-related outcome measures (e.g. DRPs, number of drugs, adverse events) is suboptimal, as these outcome measures only focus on the disadvantages of drug treatment. Furthermore the outcome measures used are often heterogeneous, as for each outcome a different set of outcome measures is used per trial. This limits the ability to draw robust conclusions. Standardization of outcome measures and time of follow-up should be applied in order to increase the ability to compare the results of trials assessing the effect of medication review. For instance, as one of the aims of the intervention is to improve the quality of life of patients, a standard set of quality of life scores (e.g. EQ-5D and SF-36) should be defined and subsequently used in future research to measure the effect of medication review on quality of life.

In the meantime, it is also conceivable that even when medication review is operationalized and/or investigated as described above, it is not effective on clinical outcomes, economical outcomes and quality of life. A possible explanation is that medication review is a cross-sectional intervention at an arbitrary moment during patient’s drug therapy. However, it might be assumed that at specific moments of drug therapy (e.g. when drugs are started, adapted or stopped) the risk for preventable drug-related problems causing negative clinical outcomes is higher. These specific high-risk moments seem to be the best occasion to apply medication optimization in order to prevent clinically relevant drug-related problems. It can therefore be suggested to redesign the cross-sectional medication review to longitudinal medication therapy management, directly from the start of a drug, targeting at specific risk moments [72]. Furthermore a more integral approach of pharmaceutical care will give room for medication improvement strategies to shift from a system repairing overdue maintenance to a more individualized approach. Problems related to prescribing according to general guidelines should be solved by means of population based interventions like for instance clinical rules. Other interventions should be developed to address issues related to a patient’s use of medication in the context of his medical condition. For instance individualized medication coaching consults with non-adherent patients or patients experiencing drug-related problems or adverse events.

A couple of limitations are associated with this systematic review. In order to provide a broad overview on the literature about the effect of medication review, no inclusion criteria were applied with regard to outcome measures. Consequently, in the best evidence syntheses, both trials using a specific outcome measure as primary outcome measure and trials using the outcome measure as secondary outcome measure were included. This possibly leads to underpowered trials being part of the best evidence synthesis (BES). However, large trials (with more power) have more impact in the BES. Furthermore, in the best evidence synthesis, it is theoretically possible that a large trial with a high risk of bias has decisive impact on both the overall risk of bias of a set studies and the conclusion about the effect of medication review on a specific outcome measure. However, only in 1/22 best evidence syntheses would the conclusion change to effect (EQ-5D VAS), when studies with a high risk of bias with a number of intervention patients greater than the median number of intervention patients of the trials would be excluded from the best evidence synthesis. Finally a limitation might be the fact that only RCTs were included in this systematic review, although it was a deliberate choice not to include observational studies, as a randomized controlled trial is the most appropriate study design to demonstrate effect of an intervention.

Besides these limitations, some remarks can be made with regard to the robustness of the conclusions. Firstly, only 55% of the included studies were designated a low risk of bias, which results in a smaller body of evidence. In a sensitivity analysis, increasing the threshold for the risk of bias assessment to an arbitrary 2/3 of the attainable maximum score, the percentage of trials with low risk of bias decreased to 39%. For medication review trials, however, on the one hand it is reasonable to relax the threshold to some extent when it comes to blindness of the patient, care provider and outcome assessor. On the other hand this may lead to an overestimation of positive findings of assessor dependent outcome measures, for instance when a non-blinded assessor has to assess whether an outcome is drug-dependent or not. Secondly, the variety of the included patients and settings in this systematic review should be considered. Although no exclusion criteria based on patient characteristics may have resulted in more power, this also may have led to false negative results in subgroups. In other systematic reviews, however, often no effect was found in these subgroups.

## Conclusions

Although an isolated medication review during a short term intervention period (how it is mostly operationalized in practice) has an effect on most drug-related outcomes, medication review has minimal effect on clinical outcomes, no effect on quality of life and no conclusion could be drawn about the effect on economical outcome measures. Therefore, it should be considered to stop performing cross-sectional medication reviews as standard care. It may also be considered to shift the focus of research from cross-sectional medication review to other strategies to improve the safety and (cost-)effectiveness of drug treatment. If, despite this, research on the effect of cross sectional medication review is still continued, high quality studies including high-risk patients and using relevant outcome measures should be conducted to assess if/when medication reviews can contribute to better medication use and subsequent better clinical outcomes. However, more effort should be put in the development and evaluation of other medication improvement strategies, like more individualized and longitudinal medication therapy management, targeting at specific risk moments of drug treatment and targeting at problems that patients experience themselves.

## Additional files

Additional file 1: Development of the search strategy and full electronic search. Full electronic search (MEDLINE). (DOCX 17 kb)
Additional file 2: Data not shown in the results section of the main text of the manuscript. **Table S1.** Effect of medication review on mortality. **Figure S1.** Meta-analysis of the studies assessing the effect of medication review on mortality. **Table S3.** Effect of medication review on the number of patients with hospital admissions. **Figure S2.** Meta-analysis of the studies assessing the effect of medication review on the number of patients with hospital admissions. **Table S4.** Effect of medication review on time to first hospital (re)admission. **Table S6.** Effect of medication review on the number of emergency admissions/visits. **Table S7.** Effect of medication review on the number of GP visits. **Table S8.** Effect of medication review on the number of outpatient visits. **Table S9.** Effect of medication review on the number of patients admitted to residential homes. **Figure S3.** Meta-analysis of the studies assessing the effect of medication review on the number of patients admitted to residential homes. **Table S10.** Effect of medication review on the number of falls per patient. **Table S11.** Effect of medication review on the number of patients falling. **Figure S4.** Meta-analysis of the studies assessing the effect of medication review on the number of patients falling. **Table S12.** Effect of medication review on the Barthel index. **Table S13.** Effect of medication review on the Standard Mini Mental State Examination. **Table S14.** Effect of medication review on the quality of life. **Table S15.** Effect of medication review on the number of drug-related problems. **Table S16.** Effect of medication review on the number of drug changes. **Table S17.** Effect of medication review on the number of drugs with a dosage decrease. **Table S18.** Effect of medication review on the number of drugs with a dosage increase. **Table S19.** Effect of medication review on the number of drugs. **Table S20.** Effect of medication review on drug costs. (DOCX 124 kb)
Additional file 3: Risk of bias assessment: sensitivity analysis. Sensitivity analysis regarding a more stringent cut-off point for risk of bias. (DOCX 19 kb)
Additional file 4: Best evidence synthesis: sensitivity analysis. Sensitivity analysis with regard to the impact of large trials with high risk of bias on every individual outcome measure in the best evidence synthesis. (DOCX 20 kb)

## Abbreviations

AOU: Assessment of underutilization of medication index
CI: Confidence interval
DRP: Drug related problem
GP: General practitioner
HRB: High risk of bias
LRB: Low risk of bias
MAI: Medication appropriateness index
PCNE: Pharmaceutical care network Europe
RCT: Randomized controlled trial
RR: Risk ratio

## References

[1] Beijer HJ, de Blaey CJ (2002). Hospitalisations caused by adverse drug reactions (ADR): a meta-analysis of observational studies. Pharm World Sci.

[2] Leendertse AJ, Visser D, Egberts AC, van den Bemt PM (2010). The relationship between study characteristics and the prevalence of medication-related hospitalizations: a literature review and novel analysis. Drug Saf.

[3] Al Hamid A, Ghaleb M, Aljadhey H, Aslanpour Z (2014). A systematic review of hospitalization resulting from medicine-related problems in adult patients. Br J Clin Pharmacol.

[4] Medicines optimisation: the safe and effective use of medicines to enable the best possible outcomes. http://www.nice.org.uk/guidance/NG5/chapter/1-recommendations#/medication-review. Accessed 1 Feb 2016. 26180890

[5] Multidisciplinary guideline polypharmacy in the elderly 2012 (NHG). https://www.nhg.org/sites/default/files/content/nhg_org/uploads/polyfarmacie_bij_ouderen.pdf. Accessed 1 Feb 2016.

[6] Grundsatzpapier zur Medikationsanalyse und zum Medikationsmanagement. http://www.abda.de/fileadmin/assets/Medikationsmanagement/Grundsatzpapier_MA_MM_GBAM.pdf. Accessed 1 Feb 2016.

[7] Guidance on the medicines use review service. http://www.nhsemployers.org/case-studies-and-resources/2012/09/guidance-on-the-medicines-use-review-service. Accessed 1 Feb 2016.

[8] Clinical Medication Review, A Practice Guide. http://www.cumbria.nhs.uk/ProfessionalZone/MedicinesManagement/Guidelines/MedicationReview-PracticeGuide2011.pdf. Accessed 1 Feb 2016.

[9] Shaw J, Seal R, Pilling M. Room for review: a guide to medication review 2002. http://www.webarchive.org.uk/wayback/archive/20140627113046/http://www.npc.nhs.uk/review_medicines/intro/resources/room_for_review.pdf. Accessed 1 Feb 2016.

[10] Clyne W, Blenkinsopp A, Seal R. A guide to medication review National Prescribing Centre 2008. http://www.webarchive.org.uk/wayback/archive/20140627113046/http://www.npc.nhs.uk/review_medicines/intro/resources/agtmr_web1.pdf. Accessed 1 Feb 2016.

[11] Position Paper on the PCNE definition of Medication Review 2016. http://www.pcne.org/upload/files/149_Position_Paper_on_PCNE_Medication_Review_final.pdf. Accessed 25 July 2016.

[12] Blenkinsopp A, Bond C, Raynor DK (2012). Medication reviews. Br J Clin Pharmacol.

[13] Medication Therapy Management in Pharmacy Practice. Core Elements of an MTM Service Model. http://www.pharmacist.com/sites/default/files/files/core_elements_of_an_mtm_practice.pdf. Accessed 20 July 2016. 10.1331/JAPhA.2008.08514 18595820

[14] Patterson SM, Cadogan CA, Kerse N, Cardwell CR, Bradley MC, Ryan C (2014). Interventions to improve the appropriate use of polypharmacy for older people. Cochrane Database Syst Rev.

[15] Tan EC, Stewart K, Elliott RA, George J (2014). Pharmacist services provided in general practice clinics: a systematic review and meta-analysis. Res Social Adm Pharm.

[16] Graabaek T, Kjeldsen LJ (2013). Medication reviews by clinical pharmacists at hospitals lead to improved patient outcomes: a systematic review. Basic Clin Pharmacol Toxicol.

[17] Holland R, Desborough J, Goodyer L, Hall S, Wright D, Loke YK (2008). Does pharmacist-led medication review help to reduce hospital admissions and deaths in older people? A systematic review and meta-analysis. Br J Clin Pharmacol.

[18] Christensen M, Lundh A (2016). Medication review in hospitalised patients to reduce morbidity and mortality. Cochrane Database Syst Rev.

[19] Alldred DP, Raynor DK, Hughes C, Barber N, Chen TF, Spoor P (2013). Interventions to optimise prescribing for older people in care homes. Cochrane Database Syst Rev.

[20] Wallerstedt SM, Kindblom JM, Nylen K, Samuelsson O, Strandell A (2014). Medication reviews for nursing home residents to reduce mortality and hospitalization: systematic review and meta-analysis. Br J Clin Pharmacol.

[21] Hatah E, Braund R, Tordoff J, Duffull SB (2014). A systematic review and meta-analysis of pharmacist-led fee-for-services medication review. Br J Clin Pharmacol.

[22] Hohl CM, Wickham ME, Sobolev B, Perry JJ, Sivilotti ML, Garrison S (2015). The effect of early in-hospital medication review on health outcomes: a systematic review. Br J Clin Pharmacol.

[23] Viswanathan M, Kahwati LC, Golin CE, Blalock SJ, Coker-Schwimmer E, Posey R (2015). Medication therapy management interventions in outpatient settings: a systematic review and meta-analysis. JAMA Intern Med.

[24] Liberati A, Altman DG, Tetzlaff J, Mulrow C, Gotzsche PC, Ioannidis JP (2009). The PRISMA statement for reporting systematic reviews and meta-analyses of studies that evaluate health care interventions: explanation and elaboration. PLoS Med.

[25] Shamseer L, Moher D, Clarke M, Ghersi D, Liberati A, Petticrew M (2015). Preferred reporting items for systematic review and meta-analysis protocols (PRISMA-P) 2015: elaboration and explanation. BMJ.

[26] van Tulder M, Furlan A, Bombardier C, Bouter L (2003). Updated method guidelines for systematic reviews in the cochrane collaboration back review group. Spine (Phila Pa 1976).

[27] van Tulder MW, Assendelft WJ, Koes BW, Bouter LM (1997). Method guidelines for systematic reviews in the Cochrane Collaboration Back Review Group for Spinal Disorders. Spine (Phila Pa 1976).

[28] Vriezekolk JE, van Lankveld WG, Geenen R, van den Ende CH (2011). Longitudinal association between coping and psychological distress in rheumatoid arthritis: a systematic review. Ann Rheum Dis.

[29] Zwikker HE, van den Bemt BJ, Vriezekolk JE, van den Ende CH, van Dulmen S (2014). Psychosocial predictors of non-adherence to chronic medication: systematic review of longitudinal studies. Patient Prefer Adherence.

[30] Lisby M, Bonnerup DK, Brock B, Gregersen PA, Jensen J, Larsen ML, et al. Medication review and patient outcomes in an orthopedic Department: a randomized controlled study. J Patient Saf. 2015. Epub ahead of print. 10.1097/PTS.0000000000000173 25742062

[31] Zillich AJ, Snyder ME, Frail CK, Lewis JL, Deshotels D, Dunham P (2014). A randomized, controlled pragmatic trial of telephonic medication therapy management to reduce hospitalization in home health patients. Health Serv Res.

[32] Burns A, Furniss L, Cooke J, Craig SKL, Scobie S (2000). Pharmacist medication review in nursing homes: A cost analysis. Int J Geriatr Psychopharmacol.

[33] Heselmans A (2015). Medication review by a clinical pharmacist at the transfer point from ICU to ward: A randomized controlled trial. J Clin Pharm Ther.

[34] Holland R, Lenaghan E, Harvey I, Smith R, Shepstone L, Lipp A (2005). Does home based medication review keep older people out of hospital? The HOMER randomised controlled trial. BMJ.

[35] Lenander C, Elfsson B, Danielsson B, Midlov P, Hasselstrom J (2014). Effects of a pharmacist-led structured medication review in primary care on drug-related problems and hospital admission rates: a randomized controlled trial. Scand J Prim Health Care.

[36] Lisby M, Thomsen A, Nielsen LP, Lyhne NM, Breum-Leer C, Fredberg U (2010). The effect of systematic medication review in elderly patients admitted to an acute ward of internal medicine. Basic Clin Pharmacol Toxicol.

[37] Sellors J, Kaczorowski J, Sellors C, Dolovich L, Woodward C, Willan A (2003). A randomized controlled trial of a pharmacist consultation program for family physicians and their elderly patients. CMAJ.

[38] Bond CM, Fish A, Porteous TH, Reid JP, Scott A, Antonazzo E (2007). A randomised controlled trial of the effects of note-based medication review by community pharmacists on prescribing of cardiovascular drugs in general practice. Int J Pharm Pract.

[39] Michalek C, Wehling M, Schlitzer J, Frohnhofen H (2014). Effects of “Fit fOR The Aged” (FORTA) on pharmacotherapy and clinical endpoints--a pilot randomized controlled study. Eur J Clin Pharmacol.

[40] Williams ME, Pulliam CC, Hunter R, Johnson TM, Owens JE, Kincaid J (2004). The short-term effect of interdisciplinary medication review on function and cost in ambulatory elderly people. J Am Geriatr Soc.

[41] Lim WS, Low HN, Chan SP, Chen HN, Ding YY, Tan TL (2004). Impact of a pharmacist consult clinic on a hospital-based geriatric outpatient clinic in Singapore. Ann Acad Med Singapore.

[42] Furniss L, Burns A, Craig SK, Scobie S, Cooke J, Faragher B (2000). Effects of a pharmacist’s medication review in nursing homes. Randomised controlled trial. Br J Psychiatry.

[43] Pope G, Wall N, Peters CM, O’Connor M, Saunders J, O’Sullivan C (2011). Specialist medication review does not benefit short-term outcomes and net costs in continuing-care patients. Age Ageing.

[44] Pit SW, Byles JE, Henry DA, Holt L, Hansen V, Bowman DA (2007). A quality use of medicines program for general practitioners and older people: a cluster randomised controlled trial. Med J Aust.

[45] Meredith S, Feldman P, Frey D, Giammarco L, Hall K, Arnold K (2002). Improving medication use in newly admitted home healthcare patients: a randomized controlled trial. J Am Geriatr Soc.

[46] Olsson IN, Runnamo R, Engfeldt P (2012). Drug treatment in the elderly: an intervention in primary care to enhance prescription quality and quality of life. Scand J Prim Health Care.

[47] Jameson J, VanNoord G, Vanderwoud K (1995). The impact of a pharmacotherapy consultation on the cost and outcome of medical therapy. J Fam Pract.

[48] Zermansky AG, Petty DR, Raynor DK, Lowe CJ, Freemantle N, Vail A (2002). Clinical medication review by a pharmacist of patients on repeat prescriptions in general practice: a randomised controlled trial. Health Technol Assess.

[49] Gallagher PF, O’Connor MN, O’Mahony D (2011). Prevention of potentially inappropriate prescribing for elderly patients: a randomized controlled trial using STOPP/START criteria. Clin Pharmacol Ther.

[50] Jameson JP, VanNoord GR (2001). Pharmacotherapy consultation on polypharmacy patients in ambulatory care. Ann Pharmacother.

[51] Britton ML, Lurvey PL (1991). Impact of medication profile review on prescribing in a general medicine clinic. Am J Hosp Pharm.

[52] Sellors C, Dalby DM, Howard M, Kaczorowski J, Sellors J (2001). A pharmacist consultation service in community-based family practices: a randomized, controlled trial in seniors. J Pharm Technol.

[53] Pacini M, Smith RD, Wilson EC, Holland R (2007). Home-based medication review in older people: is it cost effective?. Pharmacoeconomics.

[54] Onder G, Pedone C, Landi F, Cesari M, Della VC, Bernabei R (2002). Adverse drug reactions as cause of hospital admissions: results from the Italian Group of Pharmacoepidemiology in the Elderly (GIFA). J Am Geriatr Soc.

[55] Alkema GE, Wilber KH, Simmons WJ, Enguidanos SM, Frey D (2007). Prevalence of potential medication problems among dually eligible older adults in Medicaid waiver services. Ann Pharmacother.

[56] Bourgeois FT, Shannon MW, Valim C, Mandl KD (2010). Adverse drug events in the outpatient setting: an 11-year national analysis. Pharmacoepidemiol Drug Saf.

[57] Goulding MR (2004). Inappropriate medication prescribing for elderly ambulatory care patients. Arch Intern Med.

[58] Hu SH, Capezuti E, Foust JB, Boltz MP, Kim H (2012). Medication discrepancy and potentially inappropriate medication in older Chinese-American home-care patients after hospital discharge. Am J Geriatr Pharmacother.

[59] Laroche ML, Charmes JP, Nouaille Y, Fourrier A, Merle L (2006). Impact of hospitalisation in an acute medical geriatric unit on potentially inappropriate medication use. Drugs Aging.

[60] Leendertse AJ, Egberts AC, Stoker LJ, van den Bemt PM (2008). Frequency of and risk factors for preventable medication-related hospital admissions in the Netherlands. Arch Intern Med.

[61] Schuler J, Duckelmann C, Beindl W, Prinz E, Michalski T, Pichler M (2008). Polypharmacy and inappropriate prescribing in elderly internal-medicine patients in Austria. Wien Klin Wochenschr.

[62] Olivier P, Bertrand L, Tubery M, Lauque D, Montastruc JL, Lapeyre-Mestre M (2009). Hospitalizations because of adverse drug reactions in elderly patients admitted through the emergency department: a prospective survey. Drugs Aging.

[63] Ruiter R, Visser LE, Rodenburg EM, Trifiro G, Ziere G, Stricker BH (2012). Adverse drug reaction-related hospitalizations in persons aged 55 years and over: a population-based study in the Netherlands. Drugs Aging.

[64] Fialova D, Topinkova E, Gambassi G, Finne-Soveri H, Jonsson PV, Carpenter I (2005). Potentially inappropriate medication use among elderly home care patients in Europe. JAMA.

[65] Onder G, Landi F, Cesari M, Gambassi G, Carbonin P, Bernabei R (2003). Inappropriate medication use among hospitalized older adults in Italy: results from the Italian Group of Pharmacoepidemiology in the Elderly. Eur J Clin Pharmacol.

[66] Ruggiero C, Dell’Aquila G, Gasperini B, Onder G, Lattanzio F, Volpato S (2010). Potentially inappropriate drug prescriptions and risk of hospitalization among older, Italian, nursing home residents: the ULISSE project. Drugs Aging.

[67] Claydon-Platt K, Manias E, Dunning T (2012). Medication-related problems occurring in people with diabetes during an admission to an adult teaching hospital: a retrospective cohort study. Diabetes Res Clin Pract.

[68] O’Neil CK, Poirer TI (1998). Impact of patient knowledge, patient-pharmacist relationship, and drug perceptions on adverse drug therapy outcomes. Pharmacotherapy.

[69] Wilmer CM, Huiskes VJB, Natsch S, Rennings AJM, van den Bemt BJF, Bos JM. Drug-related problems in a clinical setting: a literature review and cross-sectional study evaluating factors to identify patients at risk. Eur J Hosp Pharm. 2015;22:229–35.

[70] De Smet PA, Denneboom W, Kramers C, Grol R (2007). A composite screening tool for medication reviews of outpatients: general issues with specific examples. Drugs Aging.

[71] Hinchliffe A. Medicines use review by community pharmacists. http://www2.nphs.wales.nhs.uk:8080/PharmaceuticalPHTDocs.nsf/($All)/49CAA20A63ADF04E802578AA00379DEF/$File/Microsoft%20Word%20-%20Medicines%20use%20review%20by%20community%20pharmacists%20v1%200%20_2_.pdf?OpenElement. Accessed 7 Sep 2016.

[72] van den Bemt BJF, Huiskes VJB. The medication therapy management pyramid shifting medication review to an integrated medication therapy management process. Eur J Hosp Pharm. 2015;22:219–21.

[73] Briggs S, Pearce R, Dilworth S, Higgins I, Hullick C, Attia J (2015). Clinical pharmacist review: a randomised controlled trial. Emerg Med Australas.

[74] Graffen M, Kennedy D, Simpson M (2004). Quality use of medicines in the rural ambulant elderly: a pilot study. Rural Remote Health.

[75] Krska J, Cromarty JA, Arris F, Jamieson D, Hansford D, Duffus PR (2001). Pharmacist-led medication review in patients over 65: a randomized, controlled trial in primary care. Age Ageing.

[76] Kwint HF, Faber A, Gussekloo J, Bouvy ML (2011). Effects of medication review on drug-related problems in patients using automated drug-dispensing systems: a pragmatic randomized controlled study. Drugs Aging.

[77] Lenaghan E, Holland R, Brooks A (2007). Home-based medication review in a high risk elderly population in primary care--the POLYMED randomised controlled trial. Age Ageing.

[78] Mannheimer B, Ulfvarson J, Eklof S, Bergqvist M, Andersen-Karlsson E, Pettersson H (2006). Drug-related problems and pharmacotherapeutic advisory intervention at a medicine clinic. Eur J Clin Pharmacol.

[79] Meyer TJ, Van KD, Marsh S, Prochazka AV (1991). Reduction of polypharmacy by feedback to clinicians. J Gen Intern Med.

[80] Milos V, Rekman E, Bondesson A, Eriksson T, Jakobsson U, Westerlund T (2013). Improving the quality of pharmacotherapy in elderly primary care patients through medication reviews: a randomised controlled study. Drugs Aging.

[81] Zermansky AG, Alldred DP, Petty DR, Raynor DK, Freemantle N, Eastaugh J (2006). Clinical medication review by a pharmacist of elderly people living in care homes--randomised controlled trial. Age Ageing.

