# Supplementary material for: Effectiveness of medication review: a systematic review and meta-analysis of randomized controlled trials
Source: BMC Fam Pract. 2017 Jan 17;18:5. doi: 10.1186/s12875-016-0577-x (PMC5240219; doi:10.1186/s12875-016-0577-x)
Supplement: Additional file 1: — Development of the search strategy and full electronic search. Full electronic search (MEDLINE). (DOCX 17 kb) [file 12875_2016_577_MOESM1_ESM.docx]

**Additional file 1. Development of the search strategy and full electronic search**

***Development of the search strategy***

As various terms are used for medication review in published articles (like medication review, medication management, drug review, pharmacist intervention etc.), terms used to describe a medication review were identified as much as possible by first selecting references based on mesh-terms and published systematic reviews. The descriptions of medication review in these references were subsequently used as text words in a text-based search strategy. Therefore, the MEDLINE selection of eligible references consisted of a three-step approach: 1) MeSH-based selection 2) Selection of references included in published systematic reviews, 3) Text-based selection (based on text words of included references from step 1 and 2).

*1) MeSH-based selection of references*

First, studies were identified in MEDLINE by using the MeSH headings "Drug Utilization Review" or "Pharmaceutical Services" to search for interventions. These were combined with both a MeSH heading and text words to search for randomized controlled trials ("Randomized Controlled Trial" [Publication Type] OR randomized controlled trial[tw] OR randomised controlled trial[tw]).

*2) Selection of references based on published systematic reviews*

The same MeSH headings to search for interventions ("Drug Utilization Review" or "Pharmaceutical Services") were combined in a second search with the MeSH headings “Review" or "Meta-Analysis" as publication type to identify systematic reviews and meta-analyses on this subject (from January 2007 through September 2015). References from these systematic reviews were independently screened by two reviewers for studies to be included in this review.

*3) Text-based selection of references*

Finally, the abstracts of the included references from step 1 and 2 of the literature search were independently screened by two reviewers for text words to develop a third search consisting of a broad range of text words to search for “interventions” and “drug use”. These text words were also combined with the MeSH heading and text words for the publication type randomized controlled trial.

In order to perform the same search in EMBASE, the MeSH headings used for the MEDLINE search were converted to subject headings and the text words were converted to the category “multi-purpose”. Subsequently, the references of all articles included were screened for eligible references. Finally, in Web of Science, the citations of all included trials were checked to identify eligible references.

***Full electronic search (MEDLINE)***

**((((((((("Drug Utilization Review"[Mesh]) OR "Pharmaceutical Services"[Mesh])) AND ((((("Randomized Controlled Trial"[Publication Type]) OR "randomized controlled trial"[tw]) OR "randomised controlled trial"[tw]) OR "randomised controlled study"[tw]) OR "randomized controlled study"[tw])))) OR ((("pharmacist review"[TW] OR "pharmacist intervention"[TW] OR "pharmacist program"[TW] OR "pharmacist assessment"[TW] OR "pharmacist management"[TW] OR "pharmacist care"[TW] OR "pharmacist consult"[TW] OR "pharmacist counselling"[TW] OR "pharmacist evaluation"[TW] OR "pharmacist reviews"[TW] OR "pharmacist interventions"[TW] OR "pharmacist programs"[TW] OR "pharmacist assessments"[TW] OR "pharmacist consults"[TW] OR "pharmacist evaluations"[TW] OR "pharmacologist review"[TW] OR "pharmacologist intervention"[TW] OR "pharmacologist program"[TW] OR "pharmacologist assessment"[TW] OR "pharmacologist management"[TW] OR "pharmacologist care"[TW] OR "pharmacologist consult"[TW] OR "pharmacologist counselling"[TW] OR "pharmacologist evaluation"[TW] OR "pharmacologist reviews"[TW] OR "pharmacologist interventions"[TW] OR "pharmacologist programs"[TW] OR "pharmacologist assessments"[TW] OR "pharmacologist consults"[TW] OR "pharmacologist evaluations"[TW] OR "drug review"[TW] OR "drug intervention"[TW] OR "drug program"[TW] OR "drug assessment"[TW] OR "drug management"[TW] OR "drug care"[TW] OR "drug consult"[TW] OR "drug counselling"[TW] OR "drug evaluation"[TW] OR "drug reviews"[TW] OR "drug interventions"[TW] OR "drug programs"[TW] OR "drug assessments"[TW] OR "drug consults"[TW] OR "drug evaluations"[TW] OR "medication review"[TW] OR "medication intervention"[TW] OR "medication program"[TW] OR "medication assessment"[TW] OR "medication management"[TW] OR "medication care"[TW] OR "medication consult"[TW] OR "medication counselling"[TW] OR "medication evaluation"[TW] OR "medication reviews"[TW] OR "medication interventions"[TW] OR "medication programs"[TW] OR "medication assessments"[TW] OR "medication consults"[TW] OR "medication evaluations"[TW] OR "medicine review"[TW] OR "medicine intervention"[TW] OR "medicine program"[TW] OR "medicine assessment"[TW] OR "medicine management"[TW] OR "medicine care"[TW] OR "medicine consult"[TW] OR "medicine counselling"[TW] OR "medicine evaluation"[TW] OR "medicine reviews"[TW] OR "medicine interventions"[TW] OR "medicine programs"[TW] OR "medicine assessments"[TW] OR "medicine consults"[TW] OR "medicine evaluations"[TW] OR "pharmaceutical review"[TW] OR "pharmaceutical intervention"[TW] OR "pharmaceutical program"[TW] OR "pharmaceutical assessment"[TW] OR "pharmaceutical management"[TW] OR "pharmaceutical care"[TW] OR "pharmaceutical consult"[TW] OR "pharmaceutical counselling"[TW] OR "pharmaceutical evaluation"[TW] OR "pharmaceutical reviews"[TW] OR "pharmaceutical interventions"[TW] OR "pharmaceutical programs"[TW] OR "pharmaceutical assessments"[TW] OR "pharmaceutical consults"[TW] OR "pharmaceutical evaluations"[TW] OR "pharmaceutic review"[TW] OR "pharmaceutic intervention"[TW] OR "pharmaceutic program"[TW] OR "pharmaceutic assessment"[TW] OR "pharmaceutic management"[TW] OR "pharmaceutic care"[TW] OR "pharmaceutic consult"[TW] OR "pharmaceutic counselling"[TW] OR "pharmaceutic evaluation"[TW] OR "pharmaceutic reviews"[TW] OR "pharmaceutic interventions"[TW] OR "pharmaceutic programs"[TW] OR "pharmaceutic assessments"[TW] OR "pharmaceutic consults"[TW] OR "pharmaceutic evaluations"[TW] OR "pharmacology review"[TW] OR "pharmacology intervention"[TW] OR "pharmacology program"[TW] OR "pharmacology assessment"[TW] OR "pharmacology management"[TW] OR "pharmacology care"[TW] OR "pharmacology consult"[TW] OR "pharmacology counselling"[TW] OR "pharmacology evaluation"[TW] OR "pharmacology reviews"[TW] OR "pharmacology interventions"[TW] OR "pharmacology programs"[TW] OR "pharmacology assessments"[TW] OR "pharmacology consults"[TW] OR "pharmacology evaluations"[TW] OR "pharmacotherapeutic review"[TW] OR "pharmacotherapeutic intervention"[TW] OR "pharmacotherapeutic program"[TW] OR "pharmacotherapeutic assessment"[TW] OR "pharmacotherapeutic management"[TW] OR "pharmacotherapeutic care"[TW] OR "pharmacotherapeutic consult"[TW] OR "pharmacotherapeutic counselling"[TW] OR "pharmacotherapeutic evaluation"[TW] OR "pharmacotherapeutic reviews"[TW] OR "pharmacotherapeutic interventions"[TW] OR "pharmacotherapeutic programs"[TW] OR "pharmacotherapeutic assessments"[TW] OR "pharmacotherapeutic consults"[TW] OR "pharmacotherapeutic evaluations"[TW] OR "pharmacotherapy review"[TW] OR "pharmacotherapy intervention"[TW] OR "pharmacotherapy program"[TW] OR "pharmacotherapy assessment"[TW] OR "pharmacotherapy management"[TW] OR "pharmacotherapy care"[TW] OR "pharmacotherapy consult"[TW] OR "pharmacotherapy counselling"[TW] OR "pharmacotherapy evaluation"[TW] OR "pharmacotherapy reviews"[TW] OR "pharmacotherapy interventions"[TW] OR "pharmacotherapy programs"[TW] OR "pharmacotherapy assessments"[TW] OR "pharmacotherapy consults"[TW] OR "pharmacotherapy evaluations"[TW] OR "pharmacy review"[TW] OR "pharmacy intervention"[TW] OR "pharmacy program"[TW] OR "pharmacy assessment"[TW] OR "pharmacy management"[TW] OR "pharmacy care"[TW] OR "pharmacy consult"[TW] OR "pharmacy counselling"[TW] OR "pharmacy evaluation"[TW] OR "pharmacy reviews"[TW] OR "pharmacy interventions"[TW] OR "pharmacy programs"[TW] OR "pharmacy assessments"[TW] OR "pharmacy consults"[TW] OR "pharmacy evaluations"[TW] OR "polypharmacy review"[TW] OR "polypharmacy intervention"[TW] OR "polypharmacy program"[TW] OR "polypharmacy assessment"[TW] OR "polypharmacy management"[TW] OR "polypharmacy care"[TW] OR "polypharmacy consult"[TW] OR "polypharmacy counselling"[TW] OR "polypharmacy evaluation"[TW] OR "polypharmacy reviews"[TW] OR "polypharmacy interventions"[TW] OR "polypharmacy programs"[TW] OR "polypharmacy assessments"[TW] OR "polypharmacy consults"[TW] OR "polypharmacy evaluations"[TW] OR "prescription review"[TW] OR "prescription intervention"[TW] OR "prescription program"[TW] OR "prescription assessment"[TW] OR "prescription management"[TW] OR "prescription care"[TW] OR "prescription consult"[TW] OR "prescription counselling"[TW] OR "prescription evaluation"[TW] OR "prescription reviews"[TW] OR "prescription interventions"[TW] OR "prescription programs"[TW] OR "prescription assessments"[TW] OR "prescription consults"[TW] OR "prescription evaluations"[TW] OR "prescribing review"[TW] OR "prescribing intervention"[TW] OR "prescribing program"[TW] OR "prescribing assessment"[TW] OR "prescribing management"[TW] OR "prescribing care"[TW] OR "prescribing consult"[TW] OR "prescribing counselling"[TW] OR "prescribing evaluation"[TW] OR "prescribing reviews"[TW] OR "prescribing interventions"[TW] OR "prescribing programs"[TW] OR "prescribing assessments"[TW] OR "prescribing consults"[TW] OR "prescribing evaluations"[TW] OR "pharmacist's review"[TW] OR "pharmacist's intervention"[TW] OR "pharmacist's program"[TW] OR "pharmacist's assessment"[TW] OR "pharmacist's management"[TW] OR "pharmacist's care"[TW] OR "pharmacist's consult"[TW] OR "pharmacist's counselling"[TW] OR "pharmacist's evaluation"[TW] OR "pharmacist's reviews"[TW] OR "pharmacist's interventions"[TW] OR "pharmacist's programs"[TW] OR "pharmacist's assessments"[TW] OR "pharmacist's consults"[TW] OR "pharmacist's evaluations"[TW] OR "pharmacists review"[TW] OR "pharmacists intervention"[TW] OR "pharmacists program"[TW] OR "pharmacists assessment"[TW] OR "pharmacists management"[TW] OR "pharmacists care"[TW] OR "pharmacists consult"[TW] OR "pharmacists counselling"[TW] OR "pharmacists evaluation"[TW] OR "pharmacists reviews"[TW] OR "pharmacists interventions"[TW] OR "pharmacists programs"[TW] OR "pharmacists assessments"[TW] OR "pharmacists consults"[TW] OR "pharmacists evaluations"[TW] OR "pharmacists' review"[TW] OR "pharmacists' intervention"[TW] OR "pharmacists' program"[TW] OR "pharmacists' assessment"[TW] OR "pharmacists' management"[TW] OR "pharmacists' care"[TW] OR "pharmacists' consult"[TW] OR "pharmacists' counselling"[TW] OR "pharmacists' evaluation"[TW] OR "pharmacists' reviews"[TW] OR "pharmacists' interventions"[TW] OR "pharmacists' programs"[TW] OR "pharmacists' assessments"[TW] OR "pharmacists' consults"[TW] OR "pharmacists' evaluations"[TW] OR "pharmacologist's review"[TW] OR "pharmacologist's intervention"[TW] OR "pharmacologist's program"[TW] OR "pharmacologist's assessment"[TW] OR "pharmacologist's management"[TW] OR "pharmacologist's care"[TW] OR "pharmacologist's consult"[TW] OR "pharmacologist's counselling"[TW] OR "pharmacologist's evaluation"[TW] OR "pharmacologist's reviews"[TW] OR "pharmacologist's interventions"[TW] OR "pharmacologist's programs"[TW] OR "pharmacologist's assessments"[TW] OR "pharmacologist's consults"[TW] OR "pharmacologist's evaluations"[TW] OR "pharmacologists review"[TW] OR "pharmacologists intervention"[TW] OR "pharmacologists program"[TW] OR "pharmacologists assessment"[TW] OR "pharmacologists management"[TW] OR "pharmacologists care"[TW] OR "pharmacologists consult"[TW] OR "pharmacologists counselling"[TW] OR "pharmacologists evaluation"[TW] OR "pharmacologists reviews"[TW] OR "pharmacologists interventions"[TW] OR "pharmacologists programs"[TW] OR "pharmacologists assessments"[TW] OR "pharmacologists consults"[TW] OR "pharmacologists evaluations"[TW] OR "pharmacologists' review"[TW] OR "pharmacologists' intervention"[TW] OR "pharmacologists' program"[TW] OR "pharmacologists' assessment"[TW] OR "pharmacologists' management"[TW] OR "pharmacologists' care"[TW] OR "pharmacologists' consult"[TW] OR "pharmacologists' counselling"[TW] OR "pharmacologists' evaluation"[TW] OR "pharmacologists' reviews"[TW] OR "pharmacologists' interventions"[TW] OR "pharmacologists' programs"[TW] OR "pharmacologists' assessments"[TW] OR "pharmacologists' consults"[TW] OR "pharmacologists' evaluations"[TW] OR "drugs review"[TW] OR "drugs intervention"[TW] OR "drugs program"[TW] OR "drugs assessment"[TW] OR "drugs management"[TW] OR "drugs care"[TW] OR "drugs consult"[TW] OR "drugs counselling"[TW] OR "drugs evaluation"[TW] OR "drugs reviews"[TW] OR "drugs interventions"[TW] OR "drugs programs"[TW] OR "drugs assessments"[TW] OR "drugs consults"[TW] OR "drugs evaluations"[TW] OR "medications review"[TW] OR "medications intervention"[TW] OR "medications program"[TW] OR "medications assessment"[TW] OR "medications management"[TW] OR "medications care"[TW] OR "medications consult"[TW] OR "medications counselling"[TW] OR "medications evaluation"[TW] OR "medications reviews"[TW] OR "medications interventions"[TW] OR "medications programs"[TW] OR "medications assessments"[TW] OR "medications consults"[TW] OR "medications evaluations"[TW] OR "medicines review"[TW] OR "medicines intervention"[TW] OR "medicines program"[TW] OR "medicines assessment"[TW] OR "medicines management"[TW] OR "medicines care"[TW] OR "medicines consult"[TW] OR "medicines counselling"[TW] OR "medicines evaluation"[TW] OR "medicines reviews"[TW] OR "medicines interventions"[TW] OR "medicines programs"[TW] OR "medicines assessments"[TW] OR "medicines consults"[TW] OR "medicines evaluations"[TW] OR "pharmaceuticals review"[TW] OR "pharmaceuticals intervention"[TW] OR "pharmaceuticals program"[TW] OR "pharmaceuticals assessment"[TW] OR "pharmaceuticals management"[TW] OR "pharmaceuticals care"[TW] OR "pharmaceuticals consult"[TW] OR "pharmaceuticals counselling"[TW] OR "pharmaceuticals evaluation"[TW] OR "pharmaceuticals reviews"[TW] OR "pharmaceuticals interventions"[TW] OR "pharmaceuticals programs"[TW] OR "pharmaceuticals assessments"[TW] OR "pharmaceuticals consults"[TW] OR "pharmaceuticals evaluations"[TW] OR "pharmaceutics review"[TW] OR "pharmaceutics intervention"[TW] OR "pharmaceutics program"[TW] OR "pharmaceutics assessment"[TW] OR "pharmaceutics management"[TW] OR "pharmaceutics care"[TW] OR "pharmaceutics consult"[TW] OR "pharmaceutics counselling"[TW] OR "pharmaceutics evaluation"[TW] OR "pharmaceutics reviews"[TW] OR "pharmaceutics interventions"[TW] OR "pharmaceutics programs"[TW] OR "pharmaceutics assessments"[TW] OR "pharmaceutics consults"[TW] OR "pharmaceutics evaluations"[TW] OR "pharmacotherapeutics review"[TW] OR "pharmacotherapeutics intervention"[TW] OR "pharmacotherapeutics program"[TW] OR "pharmacotherapeutics assessment"[TW] OR "pharmacotherapeutics management"[TW] OR "pharmacotherapeutics care"[TW] OR "pharmacotherapeutics consult"[TW] OR "pharmacotherapeutics counselling"[TW] OR "pharmacotherapeutics evaluation"[TW] OR "pharmacotherapeutics reviews"[TW] OR "pharmacotherapeutics interventions"[TW] OR "pharmacotherapeutics programs"[TW] OR "pharmacotherapeutics assessments"[TW] OR "pharmacotherapeutics consults"[TW] OR "pharmacotherapeutics evaluations"[TW] OR "pharmacies review"[TW] OR "pharmacies intervention"[TW] OR "pharmacies program"[TW] OR "pharmacies assessment"[TW] OR "pharmacies management"[TW] OR "pharmacies care"[TW] OR "pharmacies consult"[TW] OR "pharmacies counselling"[TW] OR "pharmacies evaluation"[TW] OR "pharmacies reviews"[TW] OR "pharmacies interventions"[TW] OR "pharmacies programs"[TW] OR "pharmacies assessments"[TW] OR "pharmacies consults"[TW] OR "pharmacies evaluations"[TW] OR "prescriptions review"[TW] OR "prescriptions intervention"[TW] OR "prescriptions program"[TW] OR "prescriptions assessment"[TW] OR "prescriptions management"[TW] OR "prescriptions care"[TW] OR "prescriptions consult"[TW] OR "prescriptions counselling"[TW] OR "prescriptions evaluation"[TW] OR "prescriptions reviews"[TW] OR "prescriptions interventions"[TW] OR "prescriptions programs"[TW] OR "prescriptions assessments"[TW] OR "prescriptions consults"[TW] OR "prescriptions evaluations"[TW] OR "prescribings review"[TW] OR "prescribings intervention"[TW] OR "prescribings program"[TW] OR "prescribings assessment"[TW] OR "prescribings management"[TW] OR "prescribings care"[TW] OR "prescribings consult"[TW] OR "prescribings counselling"[TW] OR "prescribings evaluation"[TW] OR "prescribings reviews"[TW] OR "prescribings interventions"[TW] OR "prescribings programs"[TW] OR "prescribings assessments"[TW] OR "prescribings consults"[TW] OR "prescribings evaluations"[TW])) AND ((((("Randomized Controlled Trial"[Publication Type]) OR "randomized controlled trial"[tw]) OR "randomised controlled trial"[tw]) OR "randomised controlled study"[tw]) OR "randomized controlled study"[tw])))**
