# Supplementary material for: Effectiveness of medication review: a systematic review and meta-analysis of randomized controlled trials
Source: BMC Fam Pract. 2017 Jan 17;18:5. doi: 10.1186/s12875-016-0577-x (PMC5240219; doi:10.1186/s12875-016-0577-x)
Supplement: Additional file 2: — Data not shown in the results section of the main text of the manuscript. Table S1. Effect of medication review on mortality. Figure S1. Meta-analysis of the studies assessing the effect of medication review on mortality. Table S3. Effect of medication review on the number of patients with hospital admissions. Figure S2. Meta-analysis of the studies assessing the effect of medication review on the number of patients with hospital admissions. Table S4. Effect of medication review on time to first hospital (re)admission. Table S6. Effect of medication review on the number of emergency admissions/visits. Table S7. Effect of medication review on the number of GP visits. Table S8. Effect of medication review on the number of outpatient visits. Table S9. Effect of medication review on the number of patients admitted to residential homes. Figure S3. Meta-analysis of the studies assessing the effect of medication review on the number of patients admitted to residential homes. Table S10. Effect of medication review on the number of falls per patient. Table S11. Effect of medication review on the number of patients falling. Figure S4. Meta-analysis of the studies assessing the effect of medication review on the number of patients falling. Table S12. Effect of medication review on the Barthel index. Table S13. Effect of medication review on the Standard Mini Mental State Examination. Table S14. Effect of medication review on the quality of life. Table S15. Effect of medication review on the number of drug-related problems. Table S16. Effect of medication review on the number of drug changes. Table S17. Effect of medication review on the number of drugs with a dosage decrease. Table S18. Effect of medication review on the number of drugs with a dosage increase. Table S19. Effect of medication review on the number of drugs. Table S20. Effect of medication review on drug costs. (DOCX 124 kb) [file 12875_2016_577_MOESM2_ESM.docx]

***Additional file 2. Data not shown in the results section of the main text of the manuscript***

***Table S1.*** Effect of medication review on mortality

| **Author** | **Follow**  **up**  **(mos.)** | **Description intervention** | | **Patient selection criteria for medication review** | | | **Intervention (n/N) (%)** | **Control (n/N) (%)** | **RR (95% CI)**  **(I^2^, p value)** | **Risk of bias** |
| --- | --- | --- | --- | --- | --- | --- | --- | --- | --- | --- |
|  |  | **HCP involvement**  **R: Medication review**  **D: Decision about clinical relevancy** | **Patient**  **Involve-ment** | **Age, years** | **Nr drugs** | **Other** |  |  |  |  |
| Burns [33] /Furniss[34]  (2000) | 4 | R: (study) pharmacist  D: multidisciplinary team | No | No | No | Living in nursing home | 4/136 (3) | 14/158 (9) | 0.33 (0.11, 0.98) | HRB |
| Gallagher [35]  (2011) | 6 | R: (research) physician, medical team  D: physician | No | ≥ 65* | No | Emergency admission* | 10/190 (5) | 14/192 (7) | 0.72 (0.33, 1.58) | LRB |
| Heselmans [37] | 0^†^ | R: Pharmacist  D: Ward physician | No | >15* | No | ICU stay of at least three consecutive day* | 22/301 | 22/299 | 0.99 (0.56, 1.75) | HRB |
| Holland [38](2005) | 6 | R: (study) pharmacist  D: pharmacist or GP | Yes | > 80* | ≥ 2* | Discharged after emergency admission to own home or warden controlled accommodation* | 49/415 (12) | 63/414 (15) | 0.78 (0.55, 1.10) | LRB |
| Lenaghan [44]  (2007) | 6 | R: study-pharmacist  D: GP and study- pharmacist | Yes | > 80* | ≥ 4* | living in own homes*; ≥ 1 of following criteria*: living alone; confused mental state, vision or hearing impairment; prescribed medicines associated with medication-related morbidity; prescribed >7 regular oral medicines | 7/69 (10) | 6/67 (9) | 1.13 (0.40, 3.19) | HRB |
| Lisby [47]  (2010) | 3 | R: Clinical pharmacist and a clinical pharmacologist  D: ward physicians | Yes | ≥ 70* | ≥ 1* | expected to be admitted for more than 24 hr* | 8/50 (16) | 5/49 (10) | 1.57 (0.55, 4.46) | LRB |
| Lisby [48]  (2015) | 3 | R: Clinical pharmacist and a clinical pharmacologist  D: Orthopedic ward physicians | Yes | ≥ 65* | ≥ 4* | nonelective admission at orthopedic ward*; expected in-hospital length of stay (LOS) of a minimum of 24 hours | 3/53 (6%) | 3/55 (5%) |  | LRB |
| Mannheimer [49]  (2006) | 6 | P: nurse and clinical pharmacologist  D: physician in charge | Yes | No | ≥ 2* | patients who had been in hospital for < 24 hr on Tue. to Fri. or for < 60 hr on Mon. before a nurse screened the computerised medical record* | 29/150 (19) | 22/150 (15) | 1.32 (0.79, 2.19) | LRB |
| Pope [56]  (2011) | 6 | R: multidisciplinary panel  D: General practitioner | No | No | No | permanent patients on the continuing-care wards | 17/110 (15) | 11/115 (10) | 1.62 (0.79, 3.29) | LRB |
| Zermansky [60]  (2001/2002) | 12 | R: Study-clinical pharmacist  D: Pharmacist or GP | Yes | ≥ 65* | ≥ 1* | No | 15/598 (3) | 25/579 (4) | 0.58 (0.31, 1.09) | LRB |
| Zermansky [61]  (2006) | 6 | R: Study-clinical pharmacist  D: GP | Yes | ≥ 65* | ≥ 1* | No | 51/331 (15) | 48/330 (15) | 1.06 (0.74, 1.52) | LRB |
| **Best evidence synthesis** | | 136/2403 (6%) of intervention patients in a trial showing effect on mortality  1844/2403 (79%) of intervention patients in a trial with a low risk of bias  Conclusion: **evidence with low risk of bias for no effect** of medication review on mortality | | | | | | | **Overall RR**  0.94 (0.76, 1.17)  (I^2^ = 22.0%, p = 0.172) | |

mos.= months; HCP= healthcare professional; RR = risk ratio; *combination of inclusion criteria (= “and”); ^†^outcome measures determined directly after discharge from hospital; ICU = intensive care unit; GP = general practitioner; hr = hours; LRB = low risk of bias; HRB = high risk of bias

***Figure 1.*** Meta-analysis of the studies assessing the effect of medication review on mortality

***Table S2.*** Effect of medication review on the total number of hospital admissions

| **Author** | **Follow**  **up**  **(mos.)** | **Description intervention** | | **Patient selection criteria for medication review** | | | **Intervention (n/N)** | **Control (n/N)** | **Significance**  **(p value)** | **Risk of bias** |
| --- | --- | --- | --- | --- | --- | --- | --- | --- | --- | --- |
|  |  | **HCP involvement**  **R: Medication review**  **D: Decision about clinical relevancy** | **Patient**  **Involve-ment** | **Age, years** | **Nr drugs** | **Other** |  |  |  |  |
| Gallagher [35]  (2011) | 6 | R: (research) physician, medical team  D: physician | No | ≥ 65* | No | Emergency admission* | 67/190 | 64/192 | 0.691 | LRB |
| Graffen  [36]  (2004) | 6 | R: Pharmacist  D: GP and patient | Yes | > 65* | ≥ 5* | Living independently*; ≥ 1 of following*: use of predefined risk drugs; > 12 doses per day; > 6 diagnoses; BMI < 22 | Nr  (no. IP:202) | Nr | ns | HRB |
| Holland  [38]  (2005) | 6 | R: (study) pharmacist  D: pharmacist or GP | Yes | > 80* | ≥ 2* | Discharged after emergency admission to own home or warden controlled accommodation* | 234/415 | 178/414 | 0.009 | LRB |
| Krska [42]  (2001) | 3 | R: Clinical pharmacist  D: GP and pharmacist | Yes | ≥ 65* | ≥ 4* | ≥ 2 chronic conditions* | 12/168 | 13/164 | ns | HRB |
| Lenaghan [44]  (2007) | 6 | R: study-pharmacist  D: GP and study- pharmacist | Yes | > 80* | ≥ 4* | living in own homes*; ≥ 1 of following criteria*: living alone; confused mental state, vision or hearing impairment; prescribed medicines associated with medication-related morbidity; prescribed >7 regular oral medicines | 21/68 | 20/66 | 0.8 | HRB |
| Lenander [45]  (2014) | 12 | R: Geriatrics pharmacist  D: GP and patient | Yes | > 65* | ≥ 5* | already scheduled for an appointment with a GP* | 128/75 | 178/66 | ns | HRB |
| Lisby [47]  (2010) | 3 | R: Clinical pharmacist and a clinical pharmacologist  D: ward physicians | Yes | ≥ 70* | ≥ 1* | expected to be admitted for more than 24 hr* | 25/50 | 29/49 | ns | LRB |
| Lisby [48]  (2015) | 3 | R: Clinical pharmacist and a clinical pharmacologist  D: Orthopedic ward physicians | Yes | ≥ 65* | ≥ 4* | nonelective admission at orthopedic ward*; expected in-hospital length of stay (LOS) of a minimum of 24 hours | 27/53 | 17/55 | 0.37 | LRB |
| Pope [56]  (2011) | 6 | R: multidisciplinary panel  D: General practitioner | No | No | No | permanent patients on the continuing-care wards | 11/110 | 6/115 | 0.213 | LRB |
| Sellors [58]  (2003) | 5 | R: Pharmacist  D: Physician | Yes | ≥ 65* | ≥ 5* | had been seen by their physician within; the past 12 months*; no evidence of cognitive impairment; could understand English. | 129/379 | 139/409 | 0.28 (ed);0.77 (all) | LRB |
| Zermansky [61]  (2006) | 6 | R: Study-clinical pharmacist  D: GP | Yes | ≥ 65* | ≥ 1* | No | 66/331 | 86/330 | 0.11 | LRB |
| **Best evidence synthesis** | | 415/2041 (20%) of intervention patients in a trial showing effect on the number of hospital admissions  1528/2041 (75%) of intervention patients in a trial with a low risk of bias  Conclusion: **evidence with low risk of bias for no effect** of medication review on the number of hospital admissions | | | | | | | | |

mos.= months; HCP = healthcare professional; *combination of inclusion criteria (= “and”); IP = intervention patients; GP = general practitioner; ns = nonsignificant BMI = body mass index; hr = hours; ed = emergency department; LRB = low risk of bias; HRB = high risk of bias

***Table S3.*** Effect of medication review on the number of patients with hospital admissions

| **Author** | **Follow**  **up**  **(mos.)** | **Description intervention** | | **Patient selection criteria for medication review** | | | **Intervention (n/N) (%)** | **Control (n/N) (%)** | **RR (95% CI)**  **(I^2^, p value)** | **Risk of bias** |
| --- | --- | --- | --- | --- | --- | --- | --- | --- | --- | --- |
|  |  | **HCP involvement**  **R: Medication review**  **D: Decision about clinical relevancy** | **Patient**  **Involve-ment** | **Age, years** | **Nr drugs** | **Other** |  |  |  |  |
| Briggs [31]  (2015) | 4 | R: Hospital pharmacist  D: GP | Yes | > 70* | > 5* | Living at home* | 277/525 | 308/496 | 0.85 (0.76, 0.94) | HRB |
| Mannheimer [49]  (2006) | 6 | P: nurse and clinical pharmacologist  D: physician in charge | Yes | No | ≥ 2* | patients who had been in hospital for < 24 hr on Tue. To Fri. or for < 60 hr on Mon. before a nurse screened the computerized medical record* | 60/150 | 53/150 | 1.13 (0.85, 1.52) | LRB |
| Zermansky [60] (2001/2002) | 12 | R: Study-clinical pharmacist  D: Pharmacist or GP | Yes | ≥ 65* | ≥ 1* | No | 110/579 | 92/550 | 1.14 (0.88, 1.46) | LRB |
| Zermansky [61]  (2006) | 6 | R: Study-clinical pharmacist  D: GP | Yes | ≥ 65* | ≥ 1* | No | 47/331 | 52/330 | 0.90 (0.63, 1.30) | LRB |
| Zilich [62] (2014) | 2 | R: Pharmacist  D: Patient, pharmacist, physician | Yes22 | No | No | All new patients admitted into Medicare’s defined 60-day home health care episode were eligible. Medicare eligibility for home health benefits requires ordering services by a physician who reviews the need for a patient’s care and certifies that the patient is homebound | 83/415 | 112/480 | 0.86 (0.67, 1.10) | LRB |
| **Best evidence synthesis** | | 525/2000 (26%) of intervention patients in a trial showing effect on the number of patients admitted to the hospital  1475/2000 (74%) of intervention patients in a trial with a low risk of bias  Conclusion: **evidence with low risk of bias for no effect** of medication review on the number of patients admitted to the hospital | | | | | | | **Overall RR**  0.94 (0.82, 1.08)  (I^2^ = 42.3%, p = 0.139) | |

mos.= months; HCP= healthcare professional; *combination of inclusion criteria (= “and”); GP = general practitioner; hr = hours; LRB = low risk of bias; HRB = high risk of bias

***Figure 2.*** Meta-analysis of the studies assessing the effect of medication review on the number of patients with hospital admissions

***Table S4.*** Effect of medication review on time to first hospital (re)admission

| **Author** | **Follow**  **up**  **(mos.)** | **Description intervention** | | **Patient selection criteria for medication review** | | | **Intervention** | | **Control** | | **Significance**  **(p value)** | **Risk of bias** |
| --- | --- | --- | --- | --- | --- | --- | --- | --- | --- | --- | --- | --- |
|  |  | **HCP involvement**  **R: Medication review**  **D: Decision about clinical relevancy** | **Patient**  **Involve-ment** | **Age, years** | **Nr drugs** | **Other** | **No. pts.** | **Time (days)** | **No. pts.** | **Time (days)** |  |  |
| Lisby [47]  (2010) | 3 | R: Clinical pharmacist and a clinical pharmacologist  D: ward physicians | Yes | ≥ 70* | ≥ 1* | expected to be admitted for more than 24 hr* | 50 | Nr | 49 | Nr | 0.92 | LRB |
| Lisby [48]  (2015) | 3 | R: Clinical pharmacist and a clinical pharmacologist  D: Orthopedic ward physicians | Yes | ≥ 65* | ≥ 4* | nonelective admission at orthopedic ward*; expected in-hospital length of stay (LOS) of a minimum of 24 hours | 53 | 76 | 55 | 78 | 0.46 | LRB |
| Zillich [62] (2014) | 2 | R: Pharmacist  D: Patient, pharmacist, physician | Yes | No | No | All new patients admitted into Medicare’s defined 60-day home health care episode were eligible. Medicare eligibility for home health benefits requires ordering services by a physician who reviews the need for a patient’s care and certifies that the patient is homebound | 415 | Nr | 480 | Nr | 0.12 | LRB |
| **Best evidence synthesis** | | 0/518 (0%) of intervention patients in a trial showing effect on the time to first (re)admission  518/518 (100%) of intervention patients in a trial with a low risk of bias  Conclusion: **evidence with low risk of bias for no effect** of medication review on the time to first (re)admission | | | | | | | | | | |

mos.= months; HCP= healthcare professional; *combination of inclusion criteria (= “and”); Nr = not reported; hr = hours; LRB = low risk of bias

***Table S5.*** Effect of medication review on the length of hospital stay (days)

| **Author** | **Follow**  **up**  **(mos.)** | **Description intervention** | | **Patient selection criteria for medication review** | | | **Intervention** | | **Control** | | **Significance**  **(p value)** | **Risk of bias** |
| --- | --- | --- | --- | --- | --- | --- | --- | --- | --- | --- | --- | --- |
|  |  | **HCP involvement**  **R: Medication review**  **D: Decision about clinical relevancy** | **Patient**  **Involve-ment** | **Age, years** | **Nr drugs** | **Other** | **No. pts.** | **Stay (days)** | **No. pts.** | **Stay (days)** |  |  |
| Briggs [31]  (2015) | 4 | R: Hospital pharmacist  D: GP | Yes | > 70* | > 5* | Living at home* | 525 | 6 | 496 | 6 | 0.87 | HRB |
| Burns [33]/Furniss [34]  (2000) | 4 | R: (study) pharmacist  D: multidisciplinary team | No | No | No | Living in nursing home | 136 | 0.55 | 158 | 1.26 | ns | HRB |
| Gallagher [35]  (2011) | 6 | R: (research) physician, medical team  D: physician | No | ≥ 65* | No | Emergency admission* | 190 | 8 | 192 | 8.5 | 0.471 | LRB |
| Heselmans [37] | 0^†^ | R: Pharmacist  D: Ward physician | No | >15* | No | ICU stay of at least three consecutive day* | 301 | 34.2 | 299 | 34.5 | ns | HRB |
| Lenander [45]  (2014) | 12 | R: Geriatrics pharmacist  D: GP and patient | Yes | > 65* | ≥ 5* | already scheduled for an appointment with a GP* | 75 | 12 | 66 | 18 | ns | HRB |
| Lisby [47]  (2010) | 3 | R: Clinical pharmacist and a clinical pharmacologist  D: ward physicians | Yes | ≥ 70* | ≥ 1* | expected to be admitted for more than 24 hr* | 50 | 10 | 49 | 9.9 | ns | LRB |
| Lisby [48]  (2015) | 3 | R: Clinical pharmacist and a clinical pharmacologist  D: Orthopedic ward physicians | Yes | ≥ 65* | ≥ 4* | nonelective admission at orthopedic ward*; expected in-hospital length of stay (LOS) of a minimum of 24 hours | 53 | 7.5 | 55 | 7 | 0.65 | LRB |
| **Best evidence synthesis** | | 0/11330 (0%) of intervention patients in a trial showing effect on the length of hospital stay  293/1330 (22%) of intervention patients in a trial with a low risk of bias  Conclusion: **evidence with a high risk of bias for no effect** of medication review on the length of hospital stay | | | | | | | | | | |

mos.= months; HCP = healthcare professional; *combination of inclusion criteria (= “and”); GP = general practitioner; ns = nonsignificant; ^†^determined directly after hospital discharge; hr = hours; LRB = low risk of bias; HRB = high risk of bias

***Table S6.*** Effect of medication review on the number of emergency admissions/visits

| **Author** | **Follow**  **up**  **(mos.)** | **Description intervention** | | **Patient selection criteria for medication review** | | | **Intervention (n/N)** | **Control (n/N)** | **Significance**  **(p value)** | **Risk of bias** |
| --- | --- | --- | --- | --- | --- | --- | --- | --- | --- | --- |
|  |  | **HCP involvement**  **R: Medication review**  **D: Decision about clinical relevancy** | **Patient**  **Involve-ment** | **Age, years** | **Nr drugs** | **Other** |  |  |  |  |
| Holland [38]  (2005) | 6 | R: (study) pharmacist  D: pharmacist or GP | Yes | > 80* | ≥ 2* | Discharged after emergency admission to own home or warden controlled accommodation* | 234/415 | 178/414 | 0.009 | LRB |
| Krska [42]  (2001) | 3 | R: Clinical pharmacist  D: GP and pharmacist | Yes | ≥ 65* | ≥ 4* | ≥ 2 chronic conditions* | 6/168 | 8/164 | ns | HRB |
| Lenaghan [44]  (2007) | 6 | R: study-pharmacist  D: GP and study- pharmacist | Yes | > 80* | ≥ 4* | living in own homes*; ≥ 1 of following criteria*: living alone; confused mental state, vision or hearing impairment; prescribed medicines associated with medication-related morbidity; prescribed >7 regular oral medicines | 21/68 | 20/66 | 0.8 | HRB |
| Lisby [47]  (2010) | 3 | R: Clinical pharmacist and a clinical pharmacologist  D: ward physicians | Yes | ≥ 70* | ≥ 1* | expected to be admitted for more than 24 hr* | 5/50 | 5/49 | ns | LRB |
| Lisby [48]  (2015) | 3 | R: Clinical pharmacist and a clinical pharmacologist  D: Orthopedic ward physicians | Yes | ≥ 65* | ≥ 4* | nonelective admission at orthopedic ward*; expected in-hospital length of stay (LOS) of a minimum of 24 hours | 11/53 | 22/55 | .01 | LRB |
| Pope [56]  (2011) | 6 | R: multidisciplinary panel  D: General practitioner | No | No | No | permanent patients on the continuing-care wards | 11/110 | 6/115 | 0.213 | LRB |
| Sellors [58]  (2003) | 5 | R: Pharmacist  D: Physician | Yes | ≥ 65* | ≥ 5* | had been seen by their physician within; the past 12 months*; no evidence of cognitive impairment; could understand English. | 76/379 | 94/409 | 0.28 | LRB |
| **Best evidence synthesis** | | 468/1243 (38%) of intervention patients in a trial showing effect on the number of emergency admissions  1007/1243 (81%) of intervention patients in a trial with a low risk of bias  Conclusion: **evidence with low risk of bias for no effect** of medication review on the number of emergency admissions | | | | | | | | |

mos.= months; HCP = healthcare professional; *combination of inclusion criteria (= “and”); GP = general practitioner; hr = hours; ns = nonsignificant; LRB = low risk of bias; HRB = high risk of bias

***Table S7.*** Effect of medication review on the number of GP visits

| **Author** | **Follow**  **up**  **(mos.)** | **Description intervention** | | **Patient selection criteria for medication review** | | | **Intervention (n/N) (%)** | **Control (n/N) (%)** | **Significance**  **(p value)** | **Risk of bias** |
| --- | --- | --- | --- | --- | --- | --- | --- | --- | --- | --- |
|  |  | **HCP involvement**  **R: Medication review**  **D: Decision about clinical relevancy** | **Patient**  **Involve-ment** | **Age, years** | **Nr drugs** | **Other** |  |  |  |  |
| Gallagher [35]  (2011) | 6 | R: (research) physician, medical team  D: physician | No | ≥ 65* | No | Emergency admission* | Nr (IP:190) | Nr | 0.063 | LRB |
| Lisby [47]  (2010) | 3 | R: Clinical pharmacist and a clinical pharmacologist  D: ward physicians | Yes | ≥ 70* | ≥ 1* | expected to be admitted for more than 24 hr* | 440/50 | 515/49 | ns | LRB |
| Lisby [48]  (2015) | 3 | R: Clinical pharmacist and a clinical pharmacologist  D: Orthopedic ward physicians | Yes | ≥ 65* | ≥ 4* | nonelective admission at orthopedic ward*; expected in-hospital length of stay (LOS) of a minimum of 24 hours | 8/53 | 8/55 | 0.97 | LRB |
| Sellors [58]  (2003) | 5 | R: Pharmacist  D: Physician | Yes | ≥ 65* | ≥ 5* | had been seen by their physician within; the past 12 months*; no evidence of cognitive impairment; could understand English. | 1956/379 | 2033/409 | 0.65 | LRB |
| Zermansky [60]  (2001/2002) | 12 | R: Study-clinical pharmacist  D: Pharmacist or GP | Yes | ≥ 65* | ≥ 1* | No | Nr (IP: 579) | Nr (IP:550) | 0.69 | LRB |
| Zermansky [61]  (2006) | 6 | R: Study-clinical pharmacist  D: GP | Yes | ≥ 65* | ≥ 1* | No | 960/331 | 924/330 | 0.5 | LRB |
| **Best evidence synthesis** | | 0/1582 (0%) of intervention patients in a trial showing effect on the number of GP visits  1529/1582 (100%) of intervention patients in a trial with a low risk of bias  Conclusion: **evidence with low risk of bias for no effect** of medication review on the number of GP visits | | | | | | | | |

mos.= months; HCP = healthcare professional; *combination of inclusion criteria (= “and”); GP = general practitioner; Nr= not reported; hr = hours; ns = nonsignificant; LRB = low risk of bias;

***Table S8.*** Effect of medication review on the number of outpatient visits

| **Author** | **Follow**  **up**  **(mos.)** | **Description intervention** | | **Patient selection criteria for medication review** | | | **Intervention (n/N) (%)** | **Control (n/N) (%)** | **Significance**  **(p value)** | **Risk of bias** |
| --- | --- | --- | --- | --- | --- | --- | --- | --- | --- | --- |
|  |  | **HCP involvement**  **R: Medication review**  **D: Decision about clinical relevancy** | **Patient**  **Involve-ment** | **Age, years** | **Nr drugs** | **Other** |  |  |  |  |
| Burns [33]/Furniss [34]  (2000) | 4 | R: (study) pharmacist  D: multidisciplinary team | No | No | No | Living in nursing home | 41/136 | 84/158 | ns | HRB |
| Lisby [47]  (2010) | 3 | R: Clinical pharmacist and a clinical pharmacologist  D: ward physicians | Yes | ≥ 70* | ≥ 1* | expected to be admitted for more than 24 hr* | 60/50 | 54/49 | ns | LRB |
| Sellors [58]  (2003) | 5 | R: Pharmacist  D: Physician | Yes | ≥ 65* | ≥ 5* | had been seen by their physician within; the past 12 months*; no evidence of cognitive impairment; could understand English. | 110/379 | 127/409 | 0.4 | LRB |
| Zermansky [60] (2001/2002) | 12 | R: Study-clinical pharmacist  D: Pharmacist or GP | Yes | ≥ 65* | ≥ 1* | No | Nr (IP: 579) | Nr (IP:550) | 0.41 | LRB |
| **Best evidence synthesis** | | 0/1144 (0%) of intervention patients in a trial showing effect on the number of outpatient visits  1008/1144 (88%) of intervention patients in a trial with a low risk of bias  Conclusion: **evidence with low risk of bias for no effect** of medication review on the number of outpatient visits | | | | | | | | |

mos.= months; HCP = healthcare professional; *combination of inclusion criteria (= “and); GP = general practitioner; Nr= not reported; hr = hours; ns = nonsignificant; LRB = low risk of bias; HRB = high risk of bias

***Table S9.*** Effect of medication review on the number of patients admitted to residential homes

| **Author** | **Follow**  **up**  **(mos.)** | **Description intervention** | | **Patient selection criteria for medication review** | | | **Intervention (n/N) (%)** | **Control (n/N) (%)** | **RR (95% CI)**  **(I^2^, p value)** | **Risk of bias** |
| --- | --- | --- | --- | --- | --- | --- | --- | --- | --- | --- |
|  |  | **HCP involvement**  **R: Medication review**  **D: Decision about clinical relevancy** | **Patient**  **Involve-ment** | **Age, years** | **Nr drugs** | **Other** |  |  |  |  |
| Briggs [31]  (2015) | 4 | R: Hospital pharmacist  D: GP | Yes | > 70* | > 5* | Living at home* | 31/525 | 25/496 | 1.17 (0.70, 1.96) | HRB |
| Holland [38]  (2005) | 6 | R: (study) pharmacist  D: pharmacist or GP | Yes | > 80* | ≥ 2* | Discharged after emergency admission to own home or warden controlled accommodation* | 21/300 | 17/285 | 1.17 (0.63, 2.18) | LRB |
| **Best evidence synthesis** | | 0/825 (0%) of intervention patients in a trial showing effect on the number of patients admitted to residential homes  300/825 (36%) of intervention patients in a trial with a low risk of bias  Conclusion: **evidence with a high risk of bias for no effect** of medication review on the number of patients admitted to residential homes | | | | | | | **Overall RR**  1.17 (0.79, 1.74)  (I^2^ = 0.0%, p = 0.997) | |

mos.= months; HCP = healthcare professional; RR= risk ratio; *combination of inclusion criteria (= “and”); GP = general practitioner; LRB = low risk of bias; HRB = high risk of bias

***Figure 3.*** Meta-analysis of the studies assessing the effect of medication review on the number of patients admitted to residential homes****

***Table S10.*** Effect of medication review on the number of falls per patient

| **Author** | **Follow**  **up**  **(mos.)** | **Description intervention** | | **Patient selection criteria for medication review** | | | **Intervention** | | | **Control** | | | **Significance**  **(p value)** | **Risk of bias** |
| --- | --- | --- | --- | --- | --- | --- | --- | --- | --- | --- | --- | --- | --- | --- |
|  |  | **HCP involvement**  **R: Medication review**  **D: Decision about clinical relevancy** | **Patient**  **Involve-ment** | **Age, years** | **Nr drugs** | **Other** | **No. pts.** | **t=0** | **t=1** | **No. pts.** | **t=0** | **t=1** |  |  |
| Burns [33]  /Furniss [34]  (2000) | 4 | R: (study) pharmacist  D: multidisciplinary team | No | No | No | Living in nursing home | 136 | Nr | Nr | 158 | Nr | Nr | ns | HRB |
| Zermansky [61]  (2006) | 6 | R: Study-clinical pharmacist  D: GP | Yes | ≥ 65* | ≥ 1* | No | 331 | 1 | 0.8 | 330 | 0.9 | 1.3 | <0.0001 | LRB |
| **Best evidence synthesis** | | 331/467 (71%) of intervention patients in a trial showing effect on the number of falls  331/467 (71%) of intervention patients in a trial with a low risk of bias  Conclusion: **evidence with low risk of bias for effect** of medication review on the number of falls: medication review decreases the number of falls | | | | | | | | | | | | |

mos.= months; HCP = healthcare professional; Nr= not reported; ns = nonsignificant; *combination of inclusion criteria (= “and”); GP = general practitioner; LRB = low risk of bias; HRB = high risk of bias

***Table S11.*** Effect of medication review on the number of patients falling

| **Author** | **Follow**  **up**  **(mos.)** | **Description intervention** | | **Patient selection criteria for medication review** | | | **Intervention** | | | **Control** | | | **RR (95% CI)**  **(I^2^, p value)** | **Risk of bias** |
| --- | --- | --- | --- | --- | --- | --- | --- | --- | --- | --- | --- | --- | --- | --- |
|  |  | **HCP involvement**  **R: Medication review**  **D: Decision about clinical relevancy** | **Patient**  **Involve-ment** | **Age, years** | **Nr drugs** | **Other** | **No. pts.** | **t=0** | **t=1** | **No. pts.** | **t=0** | **t=1** |  |  |
| Gallagher [35]  (2011) | 6 | R: (research) physician, medical team  D: physician | No | ≥ 65* | No | Emergency admission* | 190 | 32 | 11 | 192 | 44 | 16 | 0.69 (0.33, 1.46) | LRB |
| Michalek [52]  (2014) | 0^†^ | R: Physicians  D: Physicians | No | > 70* | ≥ 3* | admitted to the acute geriatric unit*, stable health condition defined as no need for intermediate or intensive care unit treatment*, had at least three diseases in need for drug treatment*. | 58 | Nr | 2 | 56 | Nr | 12 | 0.16 (0.04, 0.69) | LRB |
| Pit [[55]  (2007) | 12 | R: Doctors  D: Doctors | Yes | ≥ 65* | No | living in the community* | 350 | 86 | 70 | 309 | 100 | 94 | 0.66 (0.50, 0.86) | HRB |
| Zermansky [61]  (2006) | 6 | R: Study-clinical pharmacist  D: GP | Yes | ≥ 65* | ≥ 1* | No | 331 | 145 | 84 | 330 | 128 | 106 | 0.79 (0.62, 1.01) | LRB |
| **Best evidence synthesis** | | 408/929 (44%) of intervention patients in a trial showing effect on the number of patients falling  579/929 (62%) of intervention patients in a trial with a low risk of bias  Conclusion:  based on meta-analysis: **evidence with low risk of bias for effect** of medication review on the number of patients falling (see *figure 4*): medication review decreases the number of patients falling  based on best evidence synthesis: **evidence ( with a low risk of bias) is inconclusive** about the effect of medication review on the number of patients falling | | | | | | | | | | | **Overall RR**  0.68 (0.52, 0.90)  (I^2^ = 41.0%, p = 0.166) | |

mos.= months; HCP = healthcare professional; RR= risk ratio; Nr= not reported; *combination of inclusion criteria (= “and”); ^†^determined directly after hospital discharge

GP = general practitioner; LRB = low risk of bias; HRB = high risk of bias

***Figure 4.*** Meta-analysis of the studies assessing the effect of medication review on the number of patients falling

***Table S12.*** Effect of medication review on the Barthel index

| **Author** | **Follow**  **up**  **(mos.)** | **Description intervention** | | **Patient selection criteria for medication review** | | | **Out-come meas-ure** | **Intervention** | | **Control** | | **Significance**  **(p value)** | **Risk of bias** |
| --- | --- | --- | --- | --- | --- | --- | --- | --- | --- | --- | --- | --- | --- |
|  |  | **HCP involvement**  **R: Medication review**  **D: Decision about clinical relevancy** | **Patient**  **Involve-ment** | **Age, years** | **Nr drugs** | **Other** |  | **t=0**  **(n)** | **t=1**  **(n)** | **t=0**  **(n)** | **t=1**  **(n)** |  |  |
| Pope [56]  (2011) | 6 | R: multidisciplinary panel  D: General practitioner | No | No | No | permanent patients on the continuing-care wards | Barthel index | 5.95  (n=110) | 5.94  (n=110) | 6.75  (n=115) | 6.62  (n=115) | ns | LRB |
| Michalek [52]  (2014) | 0^†^ | R: Physicians  D: Physicians | No | > 70* | ≥ 3* | admitted to the acute geriatric unit*, stable health condition defined as no need for intermediate or intensive care unit treatment*, had at least three diseases in need for drug treatment*. | Barthel index | Nr  (n=58) | Nr  (n=58) | Nr  (n=56) | Nr  (n=56) | 0.226 | LRB |
| Zermansky[61]  (2006) | 6 | R: Study-clinical pharmacist  D: GP | Yes | ≥ 65* | ≥ 1* | No | Barthel index | 10.00  (n=331) | 9.80  (n=331) | 10.10  (n=330) | 9.30  (n=330) | 0.06 | LRB |
| **Best evidence synthesis** | | 0/499 (0%) of intervention patients in a trial showing effect on the Barthel index  499/499 (100%) of intervention patients in a trial with a low risk of bias  Conclusion:  **evidence with low risk of bias for no effect** of medication review on the Barthel index | | | | | | | | | | | |

mos.= months; HCP = healthcare professional; RR= risk ratio; ns = nonsignificant; Nr= not reported; *combination of inclusion criteria (= “and”); ^†^determined directly after hospital discharge; GP = general practitioner; LRB = low risk of bias

***Table S13.*** Effect of medication review on the Standard Mini Mental State Examination

| **Author** | **Follow**  **up**  **(mos.)** | **Description intervention** | | **Patient selection criteria for medication review** | | | **Out-come meas-ure** | **Intervention** | | **Control** | | **Significance**  **(p value)** | **Risk of bias** |
| --- | --- | --- | --- | --- | --- | --- | --- | --- | --- | --- | --- | --- | --- |
|  |  | **HCP involvement**  **R: Medication review**  **D: Decision about clinical relevancy** | **Patient**  **Involve-ment** | **Age, years** | **Nr drugs** | **Other** |  | **t=0**  **(n)** | **t=1**  **(n)** | **t=0**  **(n)** | **t=1**  **(n)** |  |  |
| Burns [33]/  Furniss [34]  (2000) | 4 | R: (study) pharmacist  D: multidisciplinary team | No | No | No | Living in nursing home | SMMSE | 13.50  (n=132) | 12.50  (n=118) | 15.50  (n=149) | 17.10  (n=116) | 0.07 | HRB |
| Zermansky [61]  (2006) | 6 | R: Study-clinical pharmacist  D: GP | Yes | ≥ 65* | ≥ 1* | No | SMMSE | 13.8  (n=331) | 13.9  (n=331) | 13.1  (n=330) | 13.8  (n=330) | 0.62 | LRB |
| **Best evidence synthesis** | | 0/449 (0%) of intervention patients in a trial showing effect on the MMSE  331/449 (74%) of intervention patients in a trial with a low risk of bias  Conclusion:  **evidence with low risk of bias for no effect** of medication review on the MMSE | | | | | | | | | | | |

mos.= months; HCP = healthcare professional; SMMSE = Standard Mini Mental State Examination; *combination of inclusion criteria (= “and”); GP = general practitioner; LRB = low risk of bias; HRB = high risk of bias

***Table S14.*** Effect of medication review on the quality of life

| **Author** | **Follow**  **up**  **(mos.)** | **Description intervention** | | **Patient selection criteria for medication review** | | | **Out-come meas-ure** | **Intervention** | | **Control** | | **Significance**  **(p value)** | **Risk of bias** |
| --- | --- | --- | --- | --- | --- | --- | --- | --- | --- | --- | --- | --- | --- |
|  |  | **HCP involvement**  **R: Medication review**  **D: Decision about clinical relevancy** | **Patient**  **Involve-ment** | **Age, years** | **Nr drugs** | **Other** |  | **t=0**  **(n)** | **t=1**  **(n)** | **t=0**  **(n)** | **t=1**  **(n)** |  |  |
| Bond [30]  (2007) | 12 | R: Pharmacist  D: GP | No | < 65 * | No | Specific conditions* | EQ-5D | Nr  (n=899) | Nr  (n=761) | Nr  (n=914) | Nr  (n=769) | ns | LRB |
| Holland [38] (2005) | 6 | R: (study) pharmacist  D: pharmacist or GP | Yes | > 80* | ≥ 2* | Discharged after emergency admission to own home or warden controlled accommodation* | EQ-5D | 0.59  (n=422) | 0.46  (n=311) | 0.63  (n=417) | 0.50  (n=288) | 0.84 | LRB |
| Lenaghan [44]  (2007) | 6 | R: study-pharmacist  D: GP and study- pharmacist | Yes | > 80* | ≥ 4* | living in own homes*; ≥ 1 of following criteria*: living alone; confused mental state, vision or hearing impairment; prescribed medicines associated with medication-related morbidity; prescribed >7 regular oral medicines | EQ-5D | 0.62  (n=68) | 0.57  (n=56) | 0.57  (n=66) | 0.56  (n=49) | 0.10 | HRB |
| Lisby [47]  (2010) | 3 | R: Clinical pharmacist and a clinical pharmacologist  D: ward physicians | Yes | ≥ 70* | ≥ 1* | expected to be admitted for more than 24 hr* | EQ-5D | Nr  (n=50) | Nr  (n=33) | Nr  (n=49) | Nr  (n=36) | ns | LRB |
| Olsson [54]  (2012) | 12 | R: study-physician  D: Family physician | Yes | ≥ 75* | ≥ 5* | living in ordinary homes* | EQ-5D | Arm B  0.65  (n=49)  Arm C  0.61  (n=48) | Arm B  0.61  (n=39)  Arm C  0.40  (n=33) | Arm B  0.61  (n=47)  Arm C  0.61  (n=47) | Arm B  0.73  (n=34)  Arm C  0.73  (n=34) | ns | HRB |
| Pit [55]  (2007) | 12 | R: Doctors  D: Doctors | Yes | ≥ 65* | No | living in the community* | EQ-5D | 0.83  (n=395) | 0.89  (n=350) | 0.78  (n=348) | 0.87  (n=309) | 0.7 | HRB |
| Krska [42]  (2001) | 3 | R: Clinical pharmacist  D: GP and pharmacist | Yes | ≥ 65* | ≥ 4* | ≥ 2 chronic conditions* | SF-36 | Nr  (n=168) | Nr  (n=168) | Nr  (n=164) | Nr  (n=164) | ns | HRB |
| Sellors [58]  (2003) | 5 | R: Pharmacist  D: Physician | Yes | ≥ 65* | ≥ 5* | had been seen by their physician within; the past 12 months*; no evidence of cognitive impairment; could understand English. | SF-36 | nr  (n=431) | nr  (n=379) | nr  (n=458) | nr  (n=409) | ns | LRB |
| Holland [38]  (2005) | 6 | R: (study) pharmacist  D: pharmacist or GP | Yes | > 80* | ≥ 2* | Discharged after emergency admission to own home or warden controlled accommodation* | EQ-5D VAS for health | 62.2  (n=404) | 54.9  (n=303) | 62.3  (n=406) | 58.8  (n=275) | 0.042 | LRB |
| Lenaghan [44]  (2007) | 6 | R: study-pharmacist  D: GP and study- pharmacist | Yes | > 80* | ≥ 4* | living in own homes*; ≥ 1 of following criteria*: living alone; confused mental state, vision or hearing impairment; prescribed medicines associated with medication-related morbidity; prescribed >7 regular oral medicines | EQ-5D VAS for health | 63.7  (n=67) | 63.8  (n=44) | 65.2  (n=64) | 68.3  (n=48) | 0.21 | HRB |
| Lisby [47]  (2010) | 3 | R: Clinical pharmacist and a clinical pharmacologist  D: ward physicians | Yes | ≥ 70* | ≥ 1* | expected to be admitted for more than 24 hr* | EQ-5D VAS for health | Nr  (n=50) | 60.9  (n=33) | Nr  (n=49) | 54.7  (n=36) | 0.31 | LRB |
| Olsson [54]  (2012) | 12 | R: study-physician  D: Family physician | Yes | ≥ 75* | ≥ 5* | living in ordinary homes* | EQ-5D VAS for health | Arm B  51  (n=49)  Arm C  51  (n=48) | Arm B  54  (n=39)  Arm C  56  (n=33) | Arm B  50  (n=47)  Arm C  50  (n=47) | Arm B  56  (n=34)  Arm C  56  (n=34) | ns | HRB |
| Pit [55]  (2007) | 12 | R: Doctors  D: Doctors | Yes | ≥ 65* | No | living in the community* | EQ-5D VAS for health | 77.0  (n=389) | 80.4  (n=346) | 73.5  (n=348) | 77.9  (n=302) | 0.54 | HRB |
| **Best evidence synthesis** | | 0/1583 (0%) of intervention patients in a trial showing effect on quality of life, measured with the **EQ-5D questionnaire**  1105/1583 (70%) of intervention patients in a trial with a low risk of bias  Conclusion:  **evidence with low risk of bias for no effect** of medication review on the quality of life measured with the **EQ-5D questionnaire**  0/547 (0%) of intervention patients in a trial showing effect on quality of life, measured with the **SF-36 questionnaire**  379/547 (69%) of intervention patients in a trial with a low risk of bias  Conclusion: **evidence with low risk of bias for no effect** of medication review on the quality of life measured with the  **SF-36 questionnaire**  303/798 (38%) of intervention patients in a trial showing effect on quality of life, measured with the **EQ-5D VAS for health**  336/798 (42%) of intervention patients in a trial with a low risk of bias  Conclusion: **evidence (with a high risk of bias) is inconclusive** about the effect of medication review on the quality of life measured with the **EQ-5D VAS for health** | | | | | | | | | | | |

mos.= months; HCP = healthcare professional; Nr = not reported; ns = nonsignificant; *combination of inclusion criteria (= “and”); GP = general practitioner; hr =hours; LRB = low risk of bias; HRB = high risk of bias

***Table S15.*** Effect of medication review on the number of drug-related problems

| **Author** | **Follow**  **up**  **(mos.)** | **Description intervention** | | **Patient selection criteria for medication review** | | | **Intervention** | | | **Control** | | | **Significance**  **(p value)** | **Risk of bias** |
| --- | --- | --- | --- | --- | --- | --- | --- | --- | --- | --- | --- | --- | --- | --- |
|  |  | **HCP involvement**  **R: Medication review**  **D: Decision about clinical relevancy** | **Patient**  **Involve-ment** | **Age, years** | **Nr drugs** | **Other** | **No. pts.** | **t=0** | **t=1** | **No. pts.** | **t=0** | **t=1** |  |  |
| Heselmans [37] (2015) | 0^†^ | R: Pharmacist  D: Ward physician | No | >15* | No | ICU stay of at least three consecutive day* | 301 | 375 | 172 | 299 | 368 | 321 | <0.001 | HRB |
| Krska [42]  (2001) | 3 | R: Clinical pharmacist  D: GP and pharmacist | Yes | ≥ 65* | ≥ 4* | ≥ 2 chronic conditions* | 168 | 1206 | 256 | 164 | 1380 | 838 | significant^‡^ | HRB |
| Kwint [43]  (2011) | 6 | R: 2 research pharmacists  D: GP and community pharmacist | No | ≥ 65* | ≥ 5* | living at home*; at least one drug had to be dispensed via an automated system* | 55 | 249 | 175 | 53 | 231 | 221 | <0.01 | LRB |
| Lenander [45]  (2014) | 12 | R: Geriatrics pharmacist  D: GP and patient | Yes | > 65* | ≥ 5* | already scheduled for an appointment with a GP* | 75 | 130 | 98 | 66 | 90 | 73 | 0.72 | HRB |
| **Best evidence synthesis** | | 524/599 (87%) of intervention patients in a trial showing effect on the number of drug-related problems  55/599 (9%) of intervention patients in a trial with a low risk of bias  Conclusion:  **evidence with a high risk of bias for effect** of medication review on the number of drug-related problems: medication review decreases the number of drug-related problems | | | | | | | | | | | | |

mos.= months; HCP = healthcare professional; *combination of inclusion criteria (= “and”); ICU= intensive care unit; GP = general practitioner; LRB = low risk of bias; HRB = high risk of bias; ^†^determined directly after hospital discharge; ^‡^no p value reported

***Table S16.*** Effect of medication review on the number of drug changes

| **Author** | **Follow**  **up**  **(mos.)** | **Description intervention** | | **Patient selection criteria for medication review** | | | **Intervention** | | **Control** | | **Significance**  **(p value)** | **Risk of bias** |
| --- | --- | --- | --- | --- | --- | --- | --- | --- | --- | --- | --- | --- |
|  |  | **HCP involvement**  **R: Medication review**  **D: Decision about clinical relevancy** | **Patient**  **Involve-ment** | **Age, years** | **Nr drugs** | **Other** | **No. pts.** | **Mean no. drug changes** | **No. pts.** | **Mean no. drug changes** |  |  |
| Kwint [43]  (2011) | 6 | R: 2 research pharmacists  D: GP and community pharmacist | No | ≥ 65* | ≥ 5* | living at home*; at least one drug had to be dispensed via an automated system* | 55 | 2.2 | 53 | 1 | 0.02 | LRB |
| Zermansky [60] (2001/2002) | 12 | R: Study-clinical pharmacist  D: Pharmacist or GP | Yes | ≥ 65* | ≥ 1* | No | 579 | 2.2 | 550 | 1.9 | 0.02 | LRB |
| Zermansky [61]  (2006) | 6 | R: Study-clinical pharmacist  D: GP | Yes | ≥ 65* | ≥ 1* | No | 331 | 3.1 | 330 | 2.4 | <0.0001 | LRB |
| **Best evidence synthesis** | | 965/965 (100%) of intervention patients in a trial showing effect on the number of drug changes  965/965 (100%) of intervention patients in a trial with a low risk of bias  Conclusion:  **evidence with low risk of bias for effect** of medication review on the number of drug changes: medication review increases the number of drug changes | | | | | | | | | | |

mos.= months; HCP = healthcare professional; *combination of inclusion criteria (= “and”); GP = general practitioner; LRB = low risk of bias; HRB = high risk of bias

***Table S17.*** Effect of medication review on the number of drugs with a dosage decrease

| **Author** | **Follow**  **up**  **(mos.)** | **Description intervention** | | **Patient selection criteria for medication review** | | | **Intervention** | | **Control** | | **Significance**  **(p value)** | **Risk of bias** |
| --- | --- | --- | --- | --- | --- | --- | --- | --- | --- | --- | --- | --- |
|  |  | **HCP involvement**  **R: Medication review**  **D: Decision about clinical relevancy** | **Patient**  **Involve-ment** | **Age, years** | **Nr drugs** | **Other** | **No. pts.** | **Mean no. drugs with dosage decrease** | **No. pts.** | **Mean no. drugs with dosage decrease** |  |  |
| Britton [32]  (1991) | 3 | R: Clinical pharmacist  D: physician (assistant) | No | No | > 5 | No | 315 | 0.09 | 257 | 0.03 | 0.006 | HRB |
| Milos [53]  (2013) | 2 | R: Clinical pharmacist  D: Physician | No | ≥ 75* | No | users of the multi-dose drug dispensing system; living in nursing homes or their own homes with municipally provided home care | 171 | 0.06 | 174 | 0 | 0.03 | LRB |
| **Best evidence synthesis** | | 486/486 (100%) of intervention patients in a trial showing effect on the number of drugs with a dosage decrease  171/486 (35%) of intervention patients in a trial with a low risk of bias  Conclusion:  **evidence with a high risk of bias for effect** of medication review on the number of drugs with a dosage decrease: medication review increases the number of drugs with a dosage decrease | | | | | | | | | | |

mos.= months; HCP = healthcare professional; *combination of inclusion criteria (= “and”); LRB = low risk of bias; HRB = high risk of bias

***Table S18.*** Effect of medication review on the number of drugs with a dosage increase

| **Author** | **Follow**  **up**  **(mos.)** | **Description intervention** | | **Patient selection criteria for medication review** | | | **Intervention** | | **Control** | | **Significance**  **(p value)** | **Risk of bias** |
| --- | --- | --- | --- | --- | --- | --- | --- | --- | --- | --- | --- | --- |
|  |  | **HCP involvement**  **R: Medication review**  **D: Decision about clinical relevancy** | **Patient**  **Involve-ment** | **Age, years** | **Nr drugs** | **Other** | **No. pts.** | **Mean no. drugs with dosage decrease** | **No. pts.** | **Mean no. drugs with dosage decrease** |  |  |
| Britton [32]  (1991) | 3 | R: Clinical pharmacist  D: physician (assistant) | No | No | > 5 | No | 315 | 0.12 | 257 | 0.1 | ns | HRB |
| Milos [53]  (2013) | 2 | R: Clinical pharmacist  D: Physician | No | ≥ 75* | No | users of the multi-dose drug dispensing system; living in nursing homes or their own homes with municipally provided home care | 171 | 0.006 | 174 | 0.006 | 0.995 | LRB |
| **Best evidence synthesis** | | 0/486(0%) of intervention patients in a trial showing effect on the number of drugs with a dosage increase  171/486 (35%) of intervention patients in a trial with a low risk of bias  Conclusion:  **evidence with a high risk of bias for no effect** of medication review on the number of drugs with a dosage increase | | | | | | | | | | |

mos.= months; HCP = healthcare professional; *combination of inclusion criteria (= “and”); ns = nonsignificant; LRB = low risk of bias; HRB = high risk of bias

***Table S19.*** Effect of medication review on the number of drugs

| **Author** | **Follow**  **up**  **(mos.)** | **Description intervention** | | | **Patient selection criteria for medication review** | | | **Out-come meas-ure** | **Intervention** | | **Control** | | **Significance**  **(p value)** | **Risk of bias** |
| --- | --- | --- | --- | --- | --- | --- | --- | --- | --- | --- | --- | --- | --- | --- |
|  |  | **HCP involvement**  **R: Medication review**  **D: Decision about clinical relevancy** | | **Patient**  **Involve-ment** | **Age, years** | **Nr drugs** | **Other** |  | **t=0** | **t=1** | **t=0** | **t=1** |  |  |
| Britton [32]  (1991) | 0* | R: Clinical pharmacist  D: physician (assistant) | | No | No | > 5 | No | Nr drugs/patient | 8.72  (n=315) | -0.21  (n=315) | 8.52  (n=257) | 0.48  (n=257) | <0.001 | HRB |
| Burns [33]/Furniss [34]  (2000) | 4 | R: (study) pharmacist  D: multidisciplinary team | | No | No | No | Living in nursing home | Mean nr of prescribed drugs | 5.1 (n=136) | 4.2  (n=132) | 4.5  (n=158) | 4.4  (n=144) | <0.05 | HRB |
| Jameson [40] (1995) | 6 | R: Clinical pharmacist  D: Physician and pharmacist | | Yes | No | ≥ 5 (see other) | ≥ 2 of following risk factors: ≥ 5 drugs; ≥ 12 daily doses; ≥ 4 medication changes last 12 mos.; >3 concurrent diseases; noncompliance; drugs requiring TDM | number of chronic prescription medications | 5.6  (n=27) | 5  (n=27) | 5.7  (n=29) | 6.2  (n=29) | 0.004 | HRB |
| Lenaghan [44]  (2007) | 6 | R: study-pharmacist  D: GP and study- pharmacist | | Yes | > 80* | ≥ 4* | living in own homes*; ≥ 1 of following criteria*: living alone; confused mental state, vision or hearing impairment; prescribed medicines associated with medication-related morbidity; prescribed >7 regular oral medicines | number of drug items prescribed | 9.01  (n=68) | 8.68  (n=59) | 9.85  (n=66) | 10.33  (n=55) | 0.03 | HRB |
| Lenander  [45](2014) | 12 | R: Geriatrics pharmacist  D: GP and patient | | Yes | > 65* | ≥ 5* | already scheduled for an appointment with a GP* | number of drugs | 8.6  (n=75) | 7.9  (n=75) | 7.4  (n=66) | 7.5  (n=66) | 0.046 | HRB |
| Lim [46]  (2004) | 2 | R: pharmacist (of a pharmacist consult clinic)  D: primary physician | | Yes | No | > 3 (see other) | ≥ 1 of following criteria: TDM required; polypharmacy (>3 drugs or >9 doses per day); non-compliance; self-administered drugs that require psychomotor skill and co-ordination; nasogastric tube feeding; >1 doctor managing care; hospitalized within the last 6 months. | Mean number of medications | nr  (n=64) | nr  (n=64) | nr  (n=62) | nr  (n=62) | 0.11 | LRB |
| Michalek [52]  (2014) | 0^†^ | R: Physicians  D: Physicians | | No | > 70* | ≥ 3* | admitted to the acute geriatric unit*, stable health condition defined as no need for intermediate or intensive care unit treatment*, had at least three diseases in need for drug treatment*. | Median number of drugs | 6  (n=58) | 8  (n=58) | 6  (n=56) | 7  (n=56) | 0.915 | LRB |
| Meyer [51]  (1991) | 12 | R: study-physician (Group III, intensive intervention)  D: Physicians and nurse practitioners | | No | No | ≥ 10 | being followed by providers at the medical center | Number of drugs | 11.6  (n=206) | 8.6  (n=206) | 11.8  (n=88) | 8.9  (n=88) | 0.230 | HRB |
| Olsson [54]  (2012) | 12 | R: study-physician  D: Family physician | | Yes | ≥ 75* | ≥ 5* | living in ordinary homes* | Nr drugs/patient | Arm B  10 (n=49)  Arm C  10  (n=50) | Arm B  11  (n=39)  Arm C  10  (n=33) | Arm B  8  (n=48)  Arm C  8  (n=48) | Arm B  9  (n=33)  Arm C  9  (n=33) | ns | HRB |
| Williams [59]  (2004) | 1.5 | R: Interdisciplinary team (consultant pharmacist, physician and nurse)  D: Primary physician | | Yes | ≥ 65* | ≥ 5* | ≥ 2 of the medications were potentially problematic drugs for common geriatric problems*; cognitively intact * | number prescription medication | 11.7  (n=57) | 10.2  (n=57) | 12.3  (n=76) | 12.2  (n=76) | 0.001 | HRB |
| Zermansky [60]  (2001/2002) | 12 | R: Study-clinical pharmacist  D: Pharmacist or GP | | Yes | ≥ 65* | ≥ 1* | No | Mean nr of repeat prescriptions over a 12 month period | 4.8  (n=596) | 5  (n=576) | 4.6  (n=577) | 5  (n=549) | 0.01 | LRB |
| Zermansky [61]  (2006) | 6 | R: Study-clinical pharmacist  D: GP | | Yes | ≥ 65* | ≥ 1* | No | number of repeat medicines per participant | 6.9  (n=331) | 6.7  (n=331) | 6.9  (n=330) | 6.9  (n=330) | 0.5 | LRB |
| **Best evidence synthesis** | |  | 1241/1972 (63%) of intervention patients in a trial showing effect on the number of drugs  1029/1972 (52%) of intervention patients in a trial with a low risk of bias  Conclusion: **evidence with low risk of bias for effect** of medication review on the number of drugs: medication review leads to a greater decrease or smaller increase of the number of drugs | | | | | | | | | | | |

mos.= months; HCP = healthcare professional; *combination of inclusion criteria (= “and”); Nr = not reported; GP = general practitioner; ns = nonsignificant; ^†^determined directly

after hospital discharge; LRB = low risk of bias; HRB = high risk of bias

***Table S20.*** Effect of medication review on drug costs

| **Author** | **Follow**  **up**  **(mos.)** | **Description intervention** | | | **Patient selection criteria for medication review** | | | **Out-come meas-ure** | **Intervention** | | **Control** | | **Significance**  **(p value)** | **Risk of bias** |
| --- | --- | --- | --- | --- | --- | --- | --- | --- | --- | --- | --- | --- | --- | --- |
|  |  | **HCP involvement**  **R: Medication review**  **D: Decision about clinical relevancy** | | **Patient**  **Involve-ment** | **Age, years** | **Nr drugs** | **Other** |  | **t=0** | **t=1** | **t=0** | **t=1** |  |  |
| Bond [30]  (2007) | 12 | R: Pharmacist  D: GP | | No | < 65 * | No | Specific conditions* | £/6mos. | Nr  (n=899) | nr  (n=761) | nr  (n=914) | nr  (n=769) | ns | LRB |
| Britton [32]  (1991) | 0^†^ | R: Clinical pharmacist  D: physician (assistant) | | No | No | > 5 | No | $ | 43.7  (n=315) | -0.6  (n=315) | 40.84  (n=257) | 3.31  (n=257) | <0.001 | HRB |
| Burns [33]/  Furniss [34]  (2000) | 4 | R: (study) pharmacist  D: multidisciplinary team | | No | No | No | Living in nursing home | $/4mos. | 254.42 (n=136) | 210.46 (n=132) | 228.04 (n=158) | 225.98 (n=144) | Significant^‡^ | HRB |
| Jameson [40]  (1995) | 6 | R: Clinical pharmacist  D: Physician and pharmacist | | Yes | No | ≥ 5 (see other) | ≥ 2 of following risk factors: ≥ 5 drugs; ≥ 12 daily doses; ≥ 4 medication changes last 12 mos.; >3 concurrent diseases; noncompliance; drugs requiring TDM | $/6 mos. | 929 (n=27) | 799 (n=27) | 889 (n=29) | 1052  (n=29) | 0.008 | HRB |
| Jameson [41] (2001) | 6 | R: Clinical pharmacist  D: GP and pharmacist | | Yes | No | ≥ 5 | No | $/6 mos. | 1593 (n=144) | 1657 (n=144) | 1582 (n=124) | 1602 (n=124) | ns | HRB |
| Krska [42]  (2001) | 3 | R: Clinical pharmacist  D: GP and pharmacist | | Yes | ≥ 65* | ≥ 4* | ≥ 2 chronic conditions* | £/month | 39.29 (n=168) | 38.83 (n=168) | 42.80 (n=164) | 42.61 (n=164) | ns | HRB |
| Williams [59]  (2004) | 1.5 | R: Interdisciplinary team (consultant pharmacist, physician and nurse)  D: Primary physician | | Yes | ≥ 65* | ≥ 5* | ≥ 2 of the medications were potentially problematic drugs for common geriatric problems*; cognitively intact * | $/month | 162.63 (n=57) | 135.72 (n=57) | 180.88 (n=76) | 174.12 (n=76) | 0.006 | HRB |
| Zermansky [60]  (2001/2002) | 12 | R: Study-clinical pharmacist  D: Pharmacist or GP | | Yes | ≥ 65* | ≥ 1* | No | £/28 days | 29.27 (n=596) | 31.07 (n=576) | 28.23 (n=577) | 34.85 (n=549) | 0.0001 | LRB |
| Zermansky [61]  (2006) | 6 | R: Study-clinical pharmacist  D: GP | | Yes | ≥ 65* | ≥ 1* | No | £/28 days | 42.91 (n=331) | 42.24 (n=331) | 41.67 (n=330) | 42.95 (n=330) | 0.41 | LRB |
| **Best evidence synthesis** | |  | 1107/2511 (44%) of intervention patients in a trial showing effect on drug costs  1668/2511 (66%) of intervention patients in a trial with a low risk of bias  Conclusion: **evidence (low risk of bias) is inconclusive** about the effect of medication review on drug costs | | | | | | | | | | | |

mos.= months; HCP = healthcare professional; *combination of inclusion criteria (= “and”); ^†^determined directly after visit; ^‡^no p value reported; TDM= therapeutic drug monitoring; GP = general practitioner; LRB = low risk of bias; HRB = high risk of bias
