# Supplementary material for: Effectiveness of medication review: a systematic review and meta-analysis of randomized controlled trials
Source: BMC Fam Pract. 2017 Jan 17;18:5. doi: 10.1186/s12875-016-0577-x (PMC5240219; doi:10.1186/s12875-016-0577-x)
Supplement: Additional file 3: — Risk of bias assessment: sensitivity analysis. Sensitivity analysis regarding a more stringent cut-off point for risk of bias. (DOCX 19 kb) [file 12875_2016_577_MOESM3_ESM.docx]

**Additional file 3. Risk of bias assessment: sensitivity analysis**

***Table S1*** Sensitivity analysis regarding a more stringent cut-off point for risk of bias

|  | **Trials changed from low risk of bias to high risk of bias** | | | | | **Percentage of intervention patients in trials showing effect** | **Risk of bias with actually used threshold for risk of bias**  **(% studies with LRB*)** | **Risk of bias with more stringent threshold for risk of bias (% studies with LRB*)** | **Conclusion with actually used threshold for risk of bias** | **Conclusion with more stringent threshold for risk of bias** |
| --- | --- | --- | --- | --- | --- | --- | --- | --- | --- | --- |
| **Outcome measure** | **Kwint** | **Lisby (2010)** | **Pope** | **Zermanksy (2001/2002)** | **Zermansky**  **(2006)** |  |  |  |  |  |
| Mortality |  | x | x | x | x | 6% | low (79%) | high (34%) | no effect | no effect |
| Total nr hospital admissions |  | x | x |  | x | 20% | low (75%) | low (51%) | no effect | no effect |
| Nr patients admitted to hospital |  |  |  | x | x | 26% | low (74%) | high (28%) | no effect | no effect |
| Time to first (re)admission |  | x |  |  |  | 0% | low (100%) | low (90%) | no effect | no effect |
| Length of hospital stay |  | x |  |  |  | 0% | high (22%) | high (18%) | no effect | no effect |
| Nr emergency admissions |  | x | x |  |  | 38% | low (81%) | low (68%) | no effect | no effect |
| Nr GP visits |  | x |  | x | x | 0% | low (100%) | high (39%) | no effect | no effect |
| Nr outpatient visits |  | x |  | x |  | 0% | low (88%) | high (33%) | no effect | no effect |
| Nr patients admitted to residential home |  |  |  |  |  | 0% | high (36%) | high (36%) | no effect | no effect |
| Nr falls per patient |  |  |  |  | x | 71% | low (71%) | high (0%) | effect | **inconclusive** |
| Nr patients falling |  |  |  |  | x | 44% | low (62%) | high (27%) | inconclusive | inconclusive |
| Barthel index |  |  | x |  | x | 0% | low (100%) | high (12%) | no effect | no effect |
| SMMSE |  |  |  |  | x | 0% | low (74%) | high (0%) | no effect | no effect |
| EQ-5D |  | x |  |  |  | 0% | low (70%) | low (67%) | no effect | no effect |
| EQ-5D VAS |  | x |  |  |  | 38% | high (42%) | high (36%) | inconclusive | inconclusive |
| SF-36 |  |  |  |  |  | 0% | low (69%) | low (69%) | no effect | no effect |
| Nr DRPs | x |  |  |  |  | 87% | high (9%) | high (0%) | effect | effect |
| Nr drug changes | x |  |  | x | x | 100% | low (100%) | high (0%) | effect | effect |
| Nr Drugs |  |  |  | x | x | 63% | low (52%) | high (6%) | inconclusive | inconclusive |
| Nr drugs with dosage decrease |  |  |  |  |  | 100% | high (35%) | high (35%) | effect | effect |
| Nr drugs with dosage increase |  |  |  |  |  | 0% | high (35%) | high (35%) | no effect | no effect |
| Drug costs |  |  |  | x | x | 44% | low (66%) | high (30%) | inconclusive | inconclusive |

LRB = Low risk of bias; x = outcome measure used in trial
