# Supplementary material for: Effectiveness of medication review: a systematic review and meta-analysis of randomized controlled trials
Source: BMC Fam Pract. 2017 Jan 17;18:5. doi: 10.1186/s12875-016-0577-x (PMC5240219; doi:10.1186/s12875-016-0577-x)
Supplement: Additional file 4: — Best evidence synthesis: sensitivity analysis. Sensitivity analysis with regard to the impact of large trials with high risk of bias on every individual outcome measure in the best evidence synthesis. (DOCX 20 kb) [file 12875_2016_577_MOESM4_ESM.docx]

**Additional file 4. Best evidence synthesis: sensitivity analysis**

***Table S1*** Sensitivity analysis with regard to the impact of large trials with high risk of bias on every individual outcome measure in the best evidence synthesis.

| **Outcome measure** | **Median number of intervention patients of trials using this outcome measure** | **Trials with a high risk of bias and number of intervention patients > median number of intervention patients of trials using this outcome measure** | **Percentage intervention patients in trials showing effect based on all included trials** | **Percentage intervention patients in trials showing effect after exclusion of large trials with high risk of bias** | **Risk of bias based on all included trials** | **Risk of bias after exclusion of large trials with high risk of bias** | **Conclusion based on all included trials** | **Conclusion after exclusion of large trials with high risk of bias** |
| --- | --- | --- | --- | --- | --- | --- | --- | --- |
| Mortality | 150 | Heselmans (301) | 6% | 7% | low (79%) | low (90%) | No effect | No effect |
| Total nr hospital admissions | 168 | Gallagher (190)  Holland (415) | 20% | 23% | low (75%) | low (83%) | No effect | No effect |
| Nr patients admitted to hospital | 415 | Briggs (525) | 26% | 0% | low (74%) | low (100%) | No effect | No effect |
| Time to first (re)admission | 518 | N/A | 0% | 0% | low (100%) | low (100%) | No effect | No effect |
| Length of hospital stay | 136 | Briggs (525)  Heselmans (301) | 0% | 0% | high (22%) | low (58%) | No effect | No effect |
| Nr emergency admissions | 110 | Krska (168) | 38% | 44% | low (81%) | low (94%) | No effect | **Inconclusive** |
| Nr GP visits | 261 | N/A | 0% | 0% | low (100%) | low (100%) | No effect | No effect |
| Nr outpatient visits | 258 | N/A | 0% | 0% | low (88%) | low (88%) | No effect | No effect |
| Nr patients admitted to residential home | 413 | Briggs (525) | 0% | 0% | high (36%) | low (100%) | No effect | No effect |
| Nr falls per patient | 234 | N/A | 71% | 71% | low (71%) | low (71%) | Effect | Effect |
| Nr patients falling | 261 | Pit (350) | 44% | 10% | low (62%) | low (100%) | Inconclusive | **No effect** |
| Barthel index | 110 | N/A | 0% | 0% | low (100%) | low (100%) | No effect | No effect |
| SMMSE | 225 | N/A | 0% | 0% | low (74%) | low (74%) | No effect | No effect |
| EQ-5D | 192 | Pit (350) | 0% | 0% | low (70%) | low (90%) | No effect | No effect |
| EQ-5D VAS | 72 | Pit (346) | 38% | 67% | high (42%) | low (74%) | Inconclusive | **Effect** |
| SF-36 | 274 | N/A | 0% | 0% | low (69%) | low (69%) | No effect | No effect |
| Nr DRPs | 122 | Heselmans (301)  Krska (168) | 87% | 42% | high (9%) | high (42%) | Effect | **Inconclusive** |
| Nr drug changes | 331 | N/A | 100% | 100% | low (100%) | low (100%) | Effect | Effect |
| Nr Drugs | 74 |  | 63% | 58% | low (52%) | low (83%) | Effect | **Inconclusive** |
| Nr drugs with dosage decrease | 243 | Britton (315) | 100% | 100% | high (35%) | low (100%) | Effect | Effect |
| Nr drugs with dosage increase | 243 | Britton (315) | 0% | 0% | high (35%) | low (100%) | No effect | No effect |
| Drug costs | 168 | Britton (315) | 44% | 36% | low (66%) | low (95%) | Inconclusive | **No effect** |
